# The Malaria Vaccine Implementation Programme reduced clinical malaria in Kenya, 2020 to 2022: Impact evaluation with predictive time-series models of routine surveillance data

**DOI:** 10.1186/s12936-026-05973-2

**Published:** 2026-06-15

**Authors:** John A. Painter, Erika Wallender, Andrew N. Hill, Mary Hamel, Rafiq Okine, Eliane Furrer, Rose Jalang’o, Kibor K. Keitany, Simon Kariuki, Isabella Nyang’au, Brian Seda, Aaron M. Samuels, Nelli Westercamp

**Affiliations:** 1 Division of Parasitic Diseases and Malaria, US Centers for Disease Control and Prevention, Atlanta, USA; 2 U.S. President’s Malaria Initiative, Malaria Branch, US Centers for Disease Control and Prevention, Atlanta, USA; 3 Epidemic Intelligence Service, US Centers for Disease Control and Prevention, Atlanta, USA; 4 World Health Organization, Geneva, Switzerland; 5 National Vaccines and Immunization Programme, Nairobi, Kenya; 6 National Malaria Control Programme, Nairobi, Kenya; 7 Centre for Global Health Research, Kenya Medical Research Institute, Kisumu, Kenya

**Keywords:** Uncomplicated malaria, Kenya, Routine surveillance data, RTS,S, Malaria vaccine, Impact evaluation

## Abstract

**Background:**

Quantifying the impact of malaria vaccines on outpatient malaria burden in children is of interest to many countries introducing either of the World Health Organization (WHO)-recommended vaccines (RTS,S/AS01 [RTS,S] or R21/Matrix-M [R21]). The cluster randomized implementation of RTS,S by the Kenya Ministry of Health during the Malaria Vaccine Implementation Programme (MVIP) provided a unique opportunity to measure the impact of RTS,S on uncomplicated malaria cases reported through routine malaria case surveillance data in western Kenya.

**Methods:**

From 23 implementing and 23 comparison subcounties in western Kenya, monthly numbers of confirmed uncomplicated malaria cases among individuals under 5 years of age (< 5y) and 5 years of age and older (≥ 5y) were extracted from the Kenya Health Management Information System, stored in a DHIS2 instance. Facilities that reported data ≥ 11 months per year from January 2015 through December 2022 (132 implementing, 139 comparison) were included in the analysis. Prevaccination data (January 2015–December 2019) were used to predict counterfactual case counts during the evaluation period (January 2020–December 2022), beginning when the first children vaccinated under MVIP became age-eligible for the third dose. Models of < 5y malaria cases included a covariate for ≥ 5y malaria cases to control for non-RTS,S malaria control measures. The impact was estimated as the difference between predicted and observed cases across implementing and comparison areas.

**Results:**

The proportion of vaccine eligible children (RTS,S doses at 6, 7, 9, and 24 months) among all children < 5y was small at the start of MVIP but was estimated to reach 60% by December 2022. During the 3-year evaluation period following dose-3 age-eligibility, < 5y malaria cases were 2.3% lower than predicted in non-implementing facilities and 13.8% lower in implementing facilities, a reduction of 11.6%. The reduction was 5.6% during the first year of follow-up, increasing to 8.2% after two years.

**Conclusion:**

This analysis revealed that routine health data, although age-aggregated to both vaccine eligible and noneligible children < 5 years, demonstrated a reduction in malaria cases throughout the evaluation period. A further reduction in cases was observed as more vaccine-eligible children comprised the under 5 population. High quality routine data can be used to monitor vaccine impact on clinical malaria.

**Supplementary Information:**

The online version contains supplementary material available at 10.1186/s12936-026-05973-2.

## Introduction

After phase III trials demonstrated that RTS,S/AS01_E_ (RTS,S), when given in an age-based schedule, reduced the risk of clinical malaria by 55% during 12 months following dose 3 and 39% during 48 months follow-up with a four dose schedule [1–4], the World Health Organization led the Malaria Vaccine Implementation Programme (MVIP) evaluation to address key knowledge gaps that could not be evaluated through clinical trials. These gaps included confirmation of vaccine safety in routine use, evaluation of the impact on severe malaria and mortality and feasibility of routine delivery within the Expanded Programmes on Immunizations [5].

In high malaria burden areas of three sub-Saharan African countries (Ghana, Kenya, and Malawi), the RTS,S malaria vaccine was introduced by the Ministries of Health via a cluster randomized controlled implementation, accompanied by rigorous evaluation of RTS,S rollout, providing the pivotal data leading to the WHO recommendation in 2021 for the malaria vaccine as a lifesaving malaria control measure for young children in malaria endemic countries [6]. With clinical trial results demonstrating efficacy against uncomplicated malaria, MVIP did not prioritize evaluating the impact of RTS,S on outpatient malaria (reduction in malaria cases), instead focusing on severe malaria and mortality. However, during MVIP, as other malaria control interventions were implemented concurrently with malaria vaccines (e.g. indoor residual spraying, insecticide-treated bednets), it became clear that understanding the real-world impact of RTS,S could provide helpful information to countries planning to introduce malaria vaccines. The cluster randomized controlled design of the MVIP was an ideal setting to investigate potential vaccine impact as a reduction in the number of malaria cases reported through routine surveillance data [7].

In this analysis, routinely collected monthly aggregate outpatient malaria surveillance data from the Kenya Health Management Information System (KHIS) were used to evaluate the impact of RTS,S on the number of outpatient malaria cases in children under 5 years of age in western Kenya. The analysis also provided an opportunity to characterize approaches to overcoming the challenges of using these data to estimate vaccine impact.

## Methods

During western Kenya’s MVIP, facilities in 23 subcounties were randomized to provide the RTS,S vaccine through the Ministry of Health (MoH) routine child immunization system (implementing areas) starting in September 2019, whereas 23 subcounties were randomized to delay vaccine introduction (comparison areas) until after completion of MVIP. The RTS,S vaccine schedule consisted of three primary doses delivered at 6, 7, and 9 months of age with a 4th dose administered at 24 months of age.[8, 9]. RTS,S was introduced in comparison areas in March 2023, following WHO’s October 2021 recommendation for widespread use of the vaccine [8, 9].

### Routine malaria data

Monthly aggregated data for the number of RTS,S doses administered and the number of reported uncomplicated confirmed malaria cases were retrieved from Kenya’s health management information system (HMIS), the DHIS2-based [10, 11] KHIS [hiskenya.org]. A confirmed malaria case was defined in KHIS as an outpatient with a positive malaria rapid diagnostic test or blood microscopy results. Malaria case numbers were available in KHIS beginning in January 2013. Monthly numbers of confirmed malaria cases were reported by health facilities (hospitals, health centers, dispensaries, etc.), and community health workers. In KHIS malaria cases were aggregated into two age groups: 0–59 months (< 5y) or 5-years and older (≥ 5y), except for cases diagnosed and reported by community health workers, which were not age-disaggregated until 2021 and were therefore excluded [1.7 million (7.9%) of 21.1 million total cases reported in MVIP areas]. The data for each age group were converted to monthly time-series data [12] uniquely identified by the year and month, health facility (DHIS2 orgUnit), data element, and age group.

### *Expected impact on* < *5y malaria cases*

The expected impact of RTS,S vaccination among all children < 5y in implementing subcounties was estimated by inferring the annual proportion of RTS,S age-eligible children in the < 5y population, the reduction in uncomplicated malaria cases associated with RTS,S over time based on vaccine efficacy data from clinical trials [3, 4, 13], and RTS,S coverage estimates (82.7% for dose 1, 78.1% for dose 2, 68.9% for dose 3, and 34.0% for dose 4) from the western Kenya MVIP feasibility surveys [8, 9]. It was assumed that the impact of RTS,S began in January 2020, when the first children < 5y were age-eligible for the 3rd RTS,S dose.

### Data preparation

Confirmed case data in each age-category were scanned for potential reporting errors via sequential algorithms to detect anomalous values. Data were first scanned for extreme values exceeding the median absolute deviation [14] and then scanned for seasonal outliers with seasonal and trend decomposition via locally estimated scatterplot smoothing (STL) [15, 16]. Anomalous values were removed from the study dataset (Supplement F: Confirmed cases).

To reduce reporting bias and optimize the time-series models, analyses were restricted to consistently reporting facilities. Consistently reporting confirmed malaria cases was defined as reporting confirmed cases (not overall reporting [17]) a minimum number of months each calendar year from a given year until completion of the study period (Supplement G: Consistently Reporting Facilities).

**Fig. 1:**
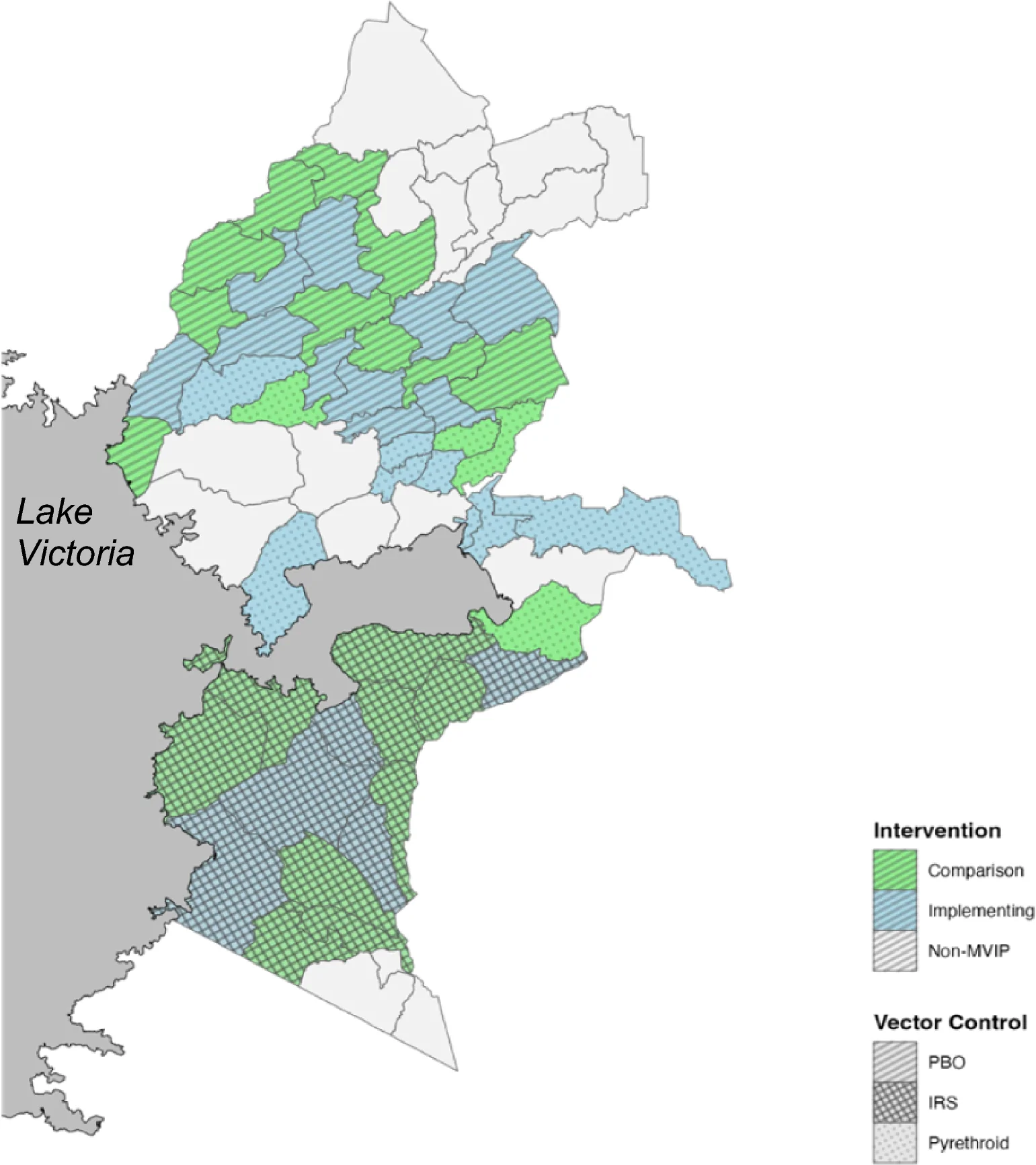
Western Kenya MVIP subcounties and vector control programs carried out independently of MVIP

Data for subcounty vector control programs carried out independently of MVIP were obtained from funder reports [18]. All subcounties in Homa Bay and Migori counties were targeted for annual indoor residual spraying (IRS) during the entire study period, and all subcounties were targeted for a mass bednet campaign in 2021 with either pyrethroid treated bednets or piperonyl-butoxide-pyrethroid synergist-treated (PBO) bednets[19] (Fig. 1) (Table 12.S, Supplement D: Enrolled health care facilities).

### Impact models

To estimate the relative impact on the number of outpatient malaria cases, reported cases were compared with predicted cases [20] during the evaluation period for each group (implementing vs comparison). The earliest vaccine effect was estimated to begin in January 2020, when the first children vaccinated under MVIP became age-eligible for the third dose. Time-series models were fit to the pre-evaluation data and counterfactual predictions for the evaluation period were generated from the best validated model, based on the out-of-sample (OOS) validation processes outlined by Linden [21] and Hyndman [22]. The data were split into a training set (up to 12-months prior to the evaluation, January 2015—December 2018) and a test set (the 12 months prior to the phased-vaccine introduction, January 2019—December 2019) [23]. Each model was fit to the training data and then used to predict cases in the evaluation period (January 2020—December 2022) [20–23].

Five model classes were evaluated via the R [24] fable packages [25]: seasonal autoregressive integrated moving average (ARIMA) [26], exponential smoothing (ETS) [27], neural network time-series forecast (NNETAR) [28], time series linear model (TSLM) [29, 30] with seasonality, and three PROPHET [31, 32] models executed with 1, 4, or 8 Fourier terms, to adjust the flexibility of model fit. The reported number of ≥ 5y cases was included as a covariate in each model except ETS, which does not allow covariates. The values for the dependent variable, < 5y cases, were log-transformed to stabilize the variance and ensure that the predicted values were nonnegative. The parameters for each primary model were selected with each package’s autofitting method. Subsequently, 120 ensemble models were created as the equally-weighted means of all the combinations[33] of the seven primary models.

For all 127 models, the predicted values were compared with actual values in the test period, and the validity was assessed with the symmetric weighted absolute percentage error (SWAPE)[23]-

$$\begin{array}{cc}& SWAPE=\frac{200\times \sum_{t=firstmonth}^{t=lastmonth}\left|reporte{d}_{t}-predicte{d}_{t}\right|}{\sum_{t=firstmonth}^{t=lastmonth}\left(\left|reporte{d}_{t}\right|+\left|predicte{d}_{t}\right|\right)}\end{array}$$

where $reporte{d}_{t}$ is the actual value at month t and where $predicte{d}_{t}$ is the predicted value at month t. The model with the lowest mean SWAPE across both the implementing and comparison groups was selected to generate the predicted numbers of cases during the intervention period.

The selected model was fit to all data prior to the intervention and used to generate a 3-year prediction for < 5y cases during the intervention period (January 2020—December 2022) based on the month and number of ≥ 5y cases in the same month. Forecasts (n = 2,000) were generated for each model; owing to the stochasticity of the time series models, the forecasts produced a range of predicted values.

Weighted percentage error (WPE)-

$$WPE=100\times \sum_{t=firstmonth}^{t=lastmonth}\left(reporte{d}_{t}-predicte{d}_{t}\right)/reporte{d}_{t}$$

was calculated for each generated forecast (n = 2,000). The estimated impact of RTS,S was calculated as the difference between the WPE of the implementing and comparison groups-

$$Impact={\mathrm{WPE}}_{Implementing}-{\mathrm{WPE}}_{Comparison}$$

yielding a distribution of estimated impacts (n = 2,000). The mean and probability that the estimated impact was greater than selected thresholds (e.g. impact > 0%, > 10%, etc.) were calculated vi the empirical cumulative distribution function (ECDF) based on the distribution of impacts.

Given the data used in this analysis, the minimum difference between the comparison and implementing groups that would result in a statistically significant WPE (95% threshold) was estimated. The WPE standard deviation for each group was assumed to be the average of the standard deviation found in this study.

### Sensitivity analysis

To investigate the sensitivity of the estimated impact to the choice of time-series model, the impact was calculated with alternative models, including the second-ranked OOS model and the top-ranked in-sample model. To evaluate the sensitivity of the results to our analytic methods, the data were reanalyzed with different modeling criteria. The main analysis used data that were cleaned (potential outliers removed) and restricted to consistently reporting facilities; as sensitivity analyses, the impact was also estimated using raw (unadjusted) data and cleaned data without the restriction to consistently reporting facilities. In addition to requiring consistently reporting facilities to report at least 11 months each year since 2015, restrictions to facilities that reported all months since 2015 were also evaluated. For each data type, the full out-of-sample and in-sample validation process was repeated to select the best-fitting model. The validation criteria were also varied to compare results when model selection was restricted to primary models (ARIMA, ETS, NNETAR, TSLM, Prophet) versus when ensemble models were also eligible for selection.

To assess the potential modifying effect of children in the comparison areas having received RTS,S at nearby implementing facilities, each comparison facility was assigned a fixed label—near or far—based on its straight-line distance to the nearest implementing facility: facilities within 5 km were categorized as near and all others as far. The overall impact of RTS,S was then estimated twice using the same approach as the main analysis: once using only near comparison facilities and once using only far comparison facilities.

### Additional details

Further details and R [24] code are available in the supplementary materials. Supplements A-H provide R packages, code, and additional details for all calculations, tables, and charts in this manuscript. All the analyses were performed in RStudio [34] running R version 4.4.1. The manuscript was compiled with Quarto [35].

## Results

### Routine malaria data

In the MVIP subcounties reporting confirmed malaria cases to KHIS, the mean number of facilities reporting malaria cases increased from 671 in 2013 to 1,301 (193%) in 2022. This represented existing facilities that successfully reported each month, not an expansion of health services.

**Fig. 2:**
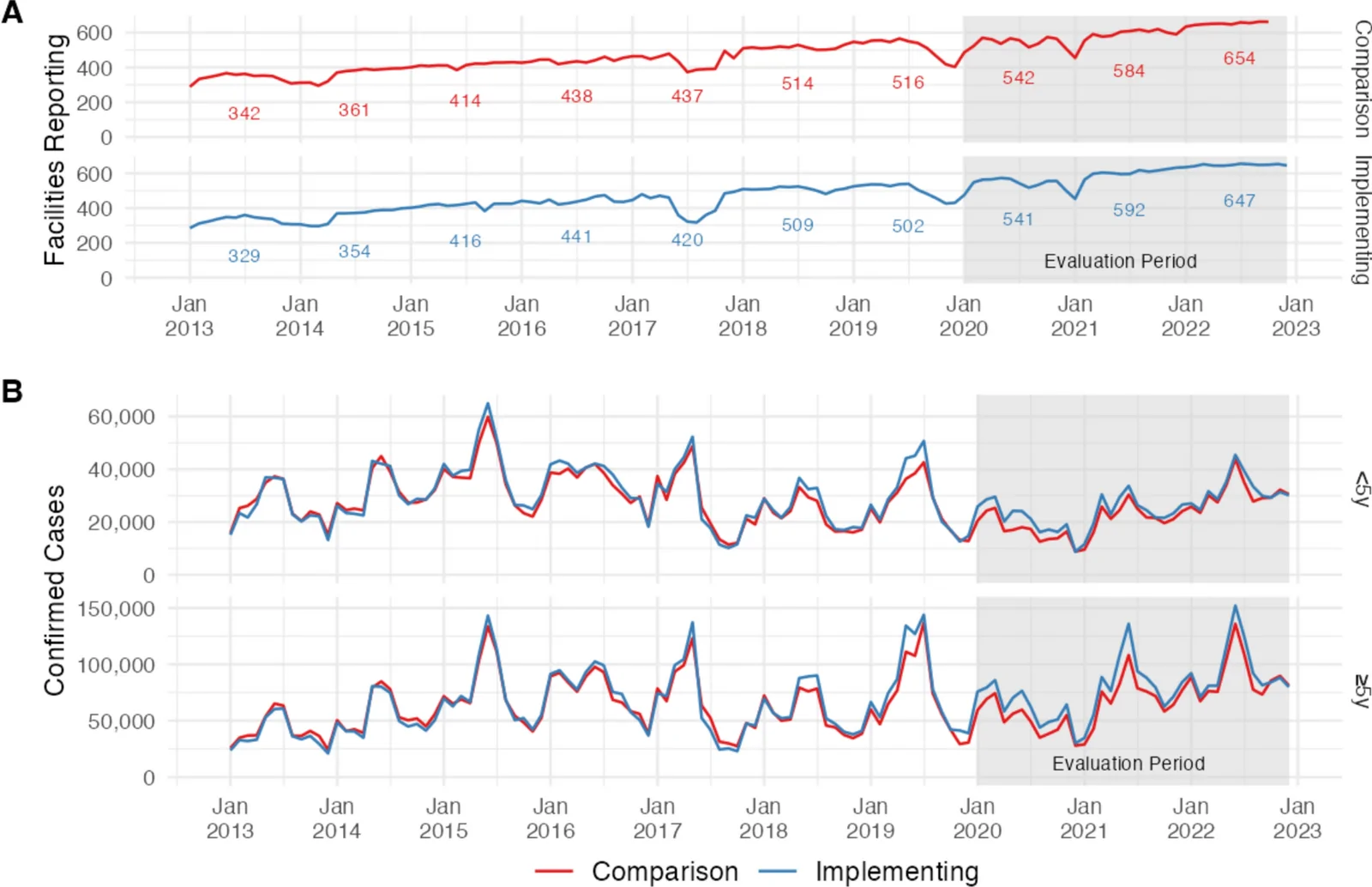
Raw (unadjusted) confirmed malaria cases in MVIP subcounties reported to KHIS. **a** Monthly number of facilities reporting confirmed malaria cases. **b** Confirmed < 5y and ≥ 5y malaria cases

**Fig. 3:**
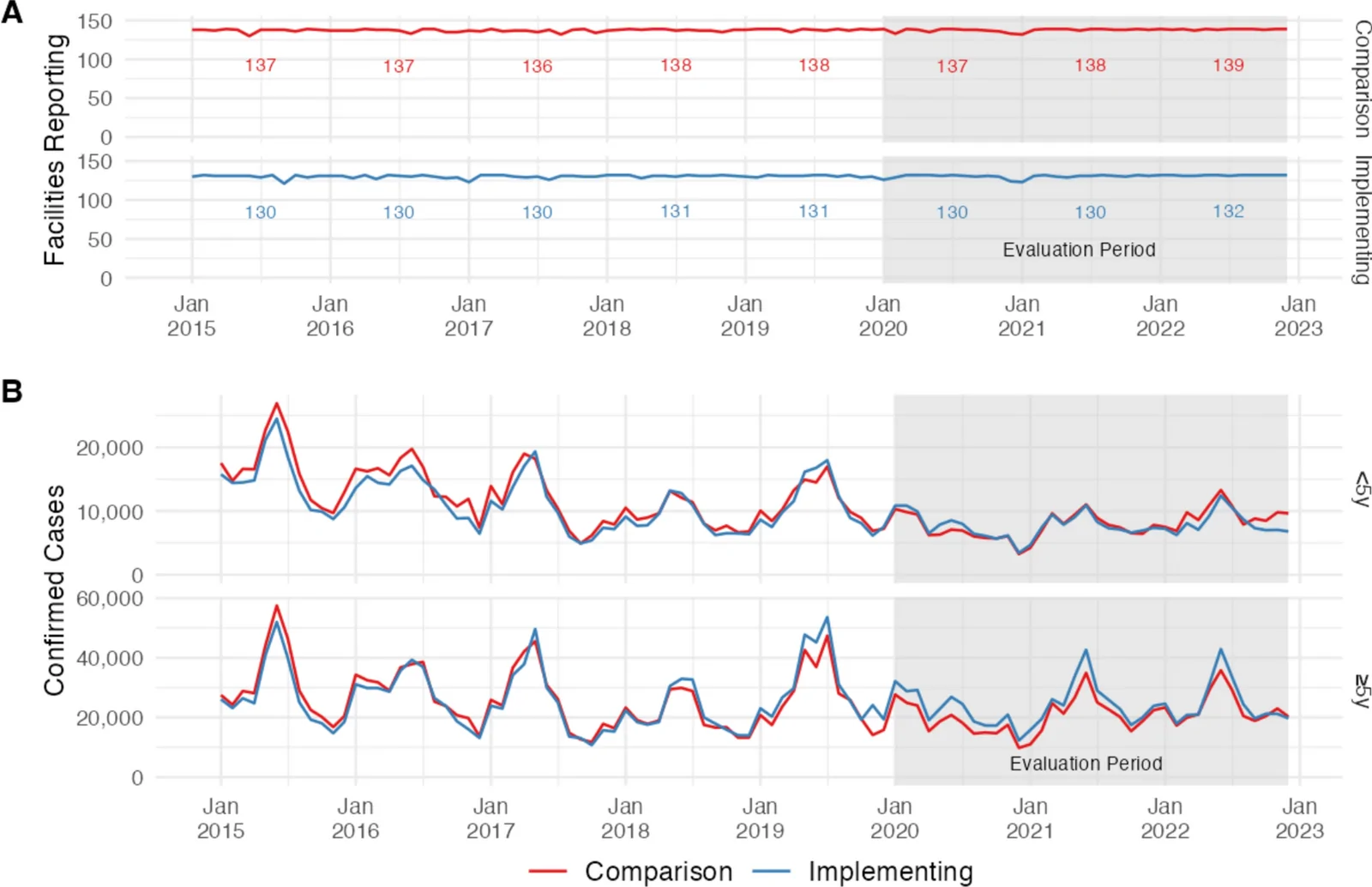
Selected facilities in MVIP subcounties reporting confirmed malaria cases. **a** Among the facilities (n = 271) included in the analysis, the monthly number reporting malaria cases. **b** Confirmed < 5 and ≥ 5 malaria cases

This trend was observed in both the implementing and comparison areas (Fig. 2). Consequently, to reduce reporting bias, we restricted the analysis to facilities that consistently reported. To allow the greatest number of health facilities to be included in the analysis while maintaining enough years to account for malaria seasonality in the model, January 2015 was selected as the starting point for the time-series models. Consequently, 271 (17.7%) facilities were included in the analysis, accounting for 1.9 million (36.1%) reported < 5y cases and 374,001 (40.3%) reported RTS,S doses administered (Fig. 3). Applying the consistently-reporting criteria with a January 2015 start date, 271 (17.7%) facilities were included in the analysis (Fig. 3), accounting for 1.9 million (36.1%) reported < 5y cases and 374,001 (40.3%) reported RTS,S doses administered.

The proportion of Ministry of Health facilities was similar between the included and excluded facilities and between each intervention group (Table 1), but there were differences in the distribution of vector control programs. Overall, a greater percentage of the comparison facilities were in areas that provided PBO-bednets in 2021 during the evaluation period (+ 10.9%), potentially reducing the number of cases in the comparison areas and biasing the results to the null. Fortuitously, this difference was only + 1.3% among the included facilities. For facilities in areas receiving annual IRS (during both the pre-evaluation and evaluation periods), the imbalance was greater overall (+ 14.4%) and among the selected facilities (+ 14.9%). Multiple facilities from each subcounty were included in the analysis, except for two implementing subcounties (Kabondo-Kasipul in Homa Bay County, and Awendo in Migori County; both implementing), which did not have facilities included because of incomplete reporting.

**Table 1:** Characteristic of MVIP health facilities

Intervention	Consistently reporting	Facilities	MOH^a^	< 5y Malaria	RTS,S Doses	PBO-nets^b^	IRS^c^
Comparison	Yes	139	60.4%	1,004,891 (18.6%)	0 (0.0%)	46.8%	33.8%
Implementing	Yes	132	59.1%	944,082 (17.5%)	374,001 (40.3%)	45.5%	18.9%
Comparison	No	642	55.0%	1,594,685 (29.6%)	0 (0.0%)	43.3%	38.8%
Implementing	No	615	56.9%	1,852,058 (34.3%)	554,645 (59.7%)	32.4%	24.5%

^a^Percent of facilities owned by Ministry of Health. Other facilities owned by 'Private', 'Mission/Faith-Based Organization', or 'Non-Governmental Organization'. Owner was not indicated for 24.8% of facilities

^b^Percent of facilities located in areas where PBO nets distributed during mass campaign in 2021

^c^Percent of facilities located in areas where IRS applied annually

Facilities are stratified by consistently reporting confirmed malaria cases (at least 11 of 12 months for each year from 2015–2022)

### RTS,S doses administered

**Fig. 4:**
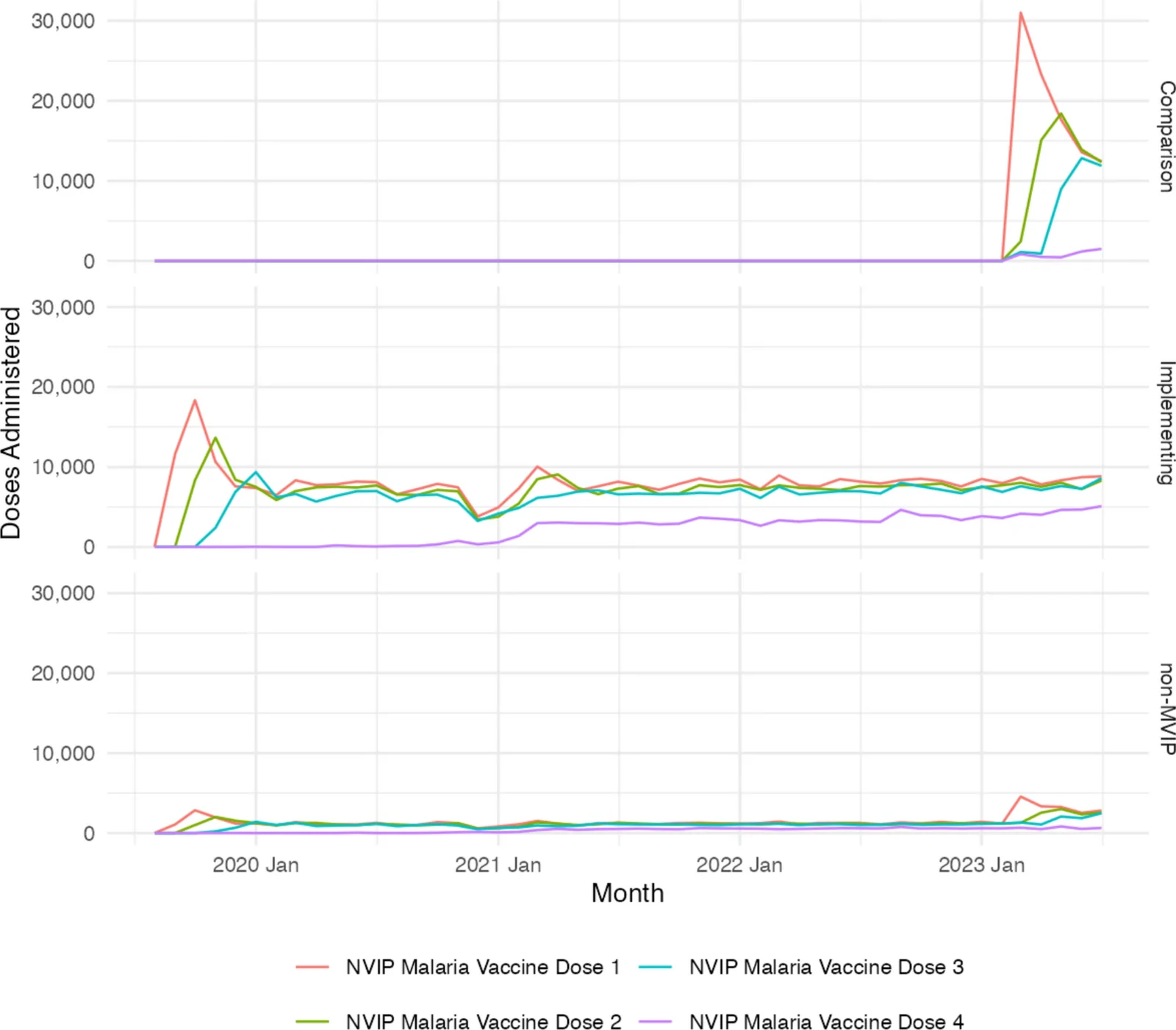
Number of RTS,S doses administered. *NVIP* National Vaccines and Immunization Programme (Kenya)

The number of first RTS,S doses administered per month peaked in quarter 4 of 2019 when the MoH expanded the age eligibility for the malaria vaccine to children up to 12 months of age (Fig. 4). Administration of the 3rd RTS,S dose per month peaked in late 2019. Although monthly vaccination rates stabilized in early 2020, marked decreases occurred between December 2020 and January 2021 during a health worker strike. This disruption likely delayed vaccination schedules, as reflected by a compensatory increase in doses administered in early 2021. Administration of the 4th dose increased over time but a considerable decrease between the 3rd and the 4th doses was observed. In the comparison areas, none of the health facilities provided the RTS,S vaccine before March 2023.

### *Expected impact on* < *5y malaria cases*

Based on the proportion of the vaccine-eligible population among < 5ys, vaccine efficacy, and vaccine coverage, the maximum expected vaccine-related reduction in < 5ys malaria cases was 8.9%, 17.8%, and 22.3% after 1, 2, and 3 years of follow-up (through December 2020, 2021, and 2022, respectively) (see Supplement B: Expected Impact).

### *Measured impact on* < *5y malaria cases*

Among the 127 models validated with pre-evaluation data, the SWAPE ranged from 7.3–68.8 (median = 37.8) for the comparison group and 5.5–68.5 (median = 40.3) for the implementing group. The ANT ensemble (ARIMA, NNETAR, and TSLM) had the lowest mean SWAPE across both groups and was selected as the best-fitting model.

During the 3-year evaluation, < 5y malaria cases were − 2.3% [− 4.4%, − 0.1%] lower than predicted in non-implementing facilities and -13.8% [-15.1%, -12.5%] lower in implementing facilities (Table 2).

**Table 2:** Estimated 36-month (2020–2022) impact of RTS,S immunizations on confirmed cases of malaria (< 5 years)

Intervention	Model	Mean	95% lower limit	95% upper limit
Comparison	ANT	− 2.3%	− 4.4%	− 0.1%
Implementing	ANT	− 13.8%	− 15.1%	− 12.5%
Cumulative Impact (1–36 months)	Implementing—Comparison	− 11.6%	− 14.2%	− 9.2%

The impact was calculated as the difference between the actual cases and the cases predicted with 2,000 forecasts from the time-series model fit to 5 years of data (2015–2019) prior to the introduction of RTS,S. Model ‘ANT’ denotes an equally weighted ensemble of models constructed with ARIMA(A), NNETAR(N), and TSLM(T) R time-series models

**Fig. 5:**
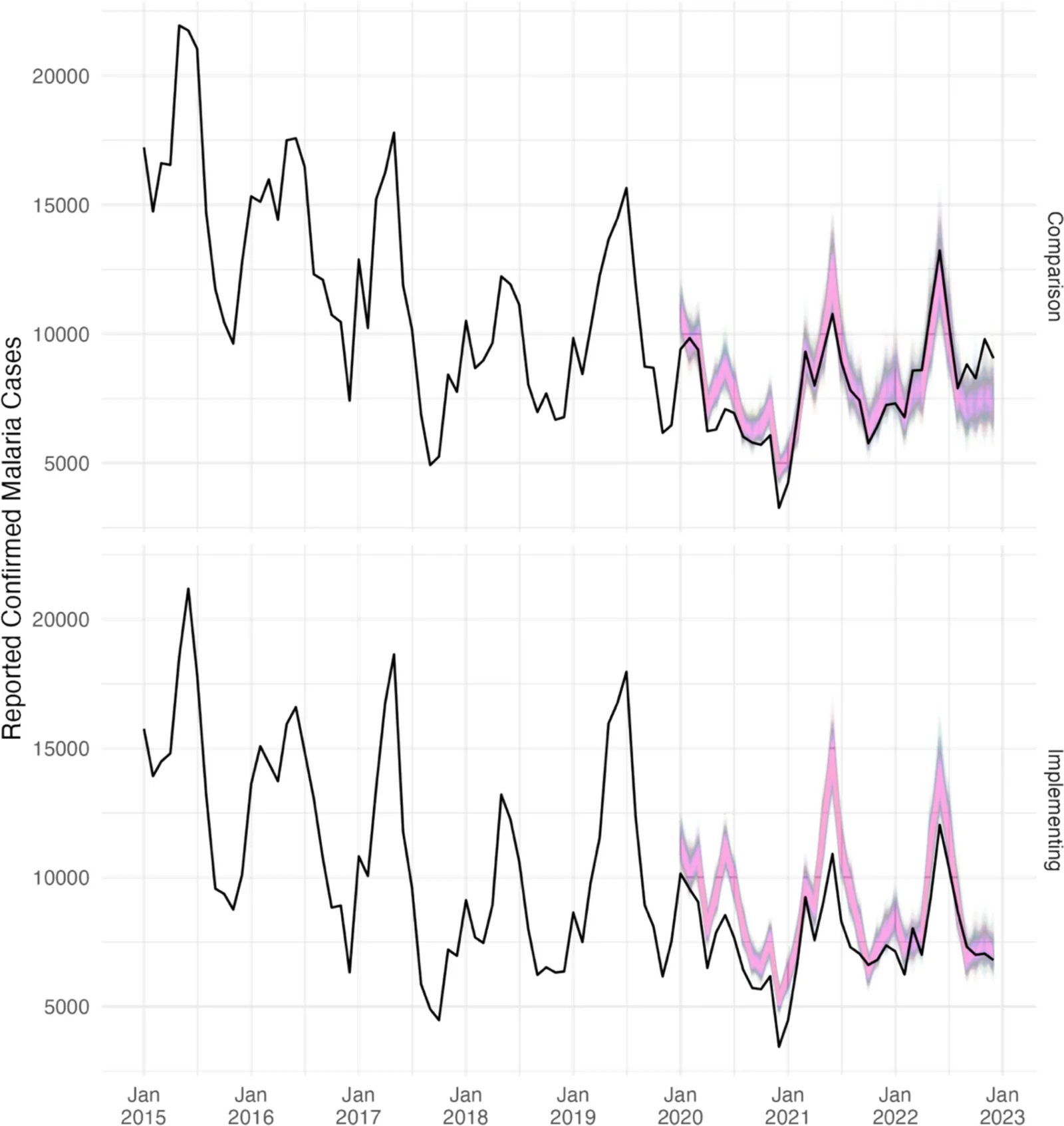
Predicted (pink) and actual cases (black) during 36-month period following RTS,S implementation

A comparison of the actual number of cases (in black) and the number of predicted cases (with 2,000 simulations, in pink) is shown in Fig. 5.

The cumulative percent change in < 5y malaria cases associated with RTS,S implementation was − 5.6% [− 8.9% 95% lower limit, − 2.7% 95% upper limit] in implementing facilities compared with non-implementing facilities after 1 year, − 8.2% [− 10.7%, − 5.8%] after 2 years, and -11.6% [-14.2%, -9.2%] after 3 years (Table 3). Given the selected data and time-series models, the smallest change that would have been statistically significant was ± 2.1%.

**Table 3:** Estimated annual cumulative impact of RTS,S immunizations on confirmed cases of malaria (< 5 years)

Evaluation Period	mean	95% lower limit	95% upper limit
1–12 months	− 5.6%	− 8.9%	− 2.7%
1–24 months	− 8.2%	− 10.7%	− 5.8%
1–36 months	− 11.6%	− 14.2%	− 9.2%

The percent difference between the actual cases and the number of predicted malaria cases without vaccination (ANT time-series model fit to 5 years of data)

### Sensitivity analysis

There was no statistically significant impact when the impact was estimated with either the raw data (− 3.2% [− 29.1%,23.9%]) or the cleaned data (− 10.6% [− 26.4%,6.3%]). When ≥ 5ys was introduced as a covariate to the cleaned and consistently reporting data, the point estimates of the percent change ranged from − 10.6% [− 13.5%, − 7.7%] to − 17.1% [− 22.6%, − 11.7%] and were all statistically significant across different model specifications (Supplement J: Alternative modeling scenarios).

Among the 126 (91%) comparison facilities included in the analysis with available geocoordinates, 43 (near) were within 5 km, and 83 (far) were ≥ 5km from one of the 549 (73%) MVIP implementing facilities with geocoordinates. Details of facility locations in Supplement I highlight the high geographic density of health facilities in western Kenya. Among near facilities, the impact was only − 6.9% [− 10.0%, − 4.3%], whereas among far facilities, the impact was − 11.7% [− 14.5%, − 9.2%], a significant difference of 4.8% [− 5.3%, − 4.3%]).

## Discussion

This analysis provides the first estimate of the RTS,S vaccine impact on outpatient malaria cases during real-world implementation in a malaria-endemic region of western Kenya. Leveraging the MVIP randomized design, analysis of routine data demonstrated population-level impact of the malaria vaccine on outpatient malaria cases with an 11.6% reduction in reported cases among children < 5ys over the three-year evaluation period (January 2020–December 2022), beginning when the first children vaccinated under MVIP became age-eligible for the primary RTS,S series (3 doses). The impact increased each year, as would be expected, as more children were vaccinated and those exposed to the 3-dose primary vaccine schedule represented a larger proportion of all children < 5y.

Attributing the reduction of clinical malaria to RTS,S was strengthened by evidence of reduced clinical cases among children in comparison areas who may have received the vaccine based on their relative proximity to implementing facilities. This analysis demonstrated that routine data can be used to measure the impact of vaccine introduction. However, it also highlights important challenges when using routine data. Aggregated data and incomplete reporting can limit the usefulness of such data. A significant impact was only evident after removing potential outliers, restricting analysis to only facilities that consistently reported confirmed cases, predicting a counterfactual from time-series models, and controlling for any differences in malaria not accounted for through randomization by including ≥ 5y malaria cases as a covariate in the models. All these strategies are important for making the most accurate estimates. Quantifying real-world reductions in malaria cases associated with the introduction of malaria vaccines can provide governments and funders useful information to estimate the incremental impact of malaria vaccines on the malaria burden and the reduced burden on the health system.

Robust clinical trials of RTS,S across geographic regions in sub-Saharan Africa demonstrated a 55% reduction in clinical malaria during the 12 months following vaccination with 3 vaccine doses and protective efficacy against clinical malaria during a median of 48 months (or approximately 4 years) of 39% with 4-doses [9]. However, when introduced through routine systems, observed vaccine effectiveness may diverge from vaccine efficacy measured in clinical trials for a number of reasons including vaccine access and coverage [4], concurrent use of other malaria control measures [8], reduced access to high quality care that is often implemented in clinical trials, and other factors impacting malaria transmission. The population-level impact on clinical malaria estimated in this analysis was less than may have been expected (22.3% during 3 years) based on vaccine efficacy and coverage estimates (69% for the 3rd dose and 34% for the 4th dose in Kenya), in part because of concurrent interventions (a higher percentage of PBO nets and IRS in comparison areas, biasing toward the null), differential care seeking behavior due to the COVID-19 pandemic, health workers strikes, and other unmeasured contextual factors. The results of this add-on study with routine data are not meant to replace carefully controlled efficacy or effectiveness studies but rather to estimate the real-world population-level impact with imperfect but available data.

Compared with more resource intensive strategies, such as sentinel sites, the use of routine surveillance data is sustainable and readily available for evaluating the impact of RTS,S. With increased use of diagnostics to confirm malaria and near real-time availability of malaria case numbers, routine surveillance data are increasingly being used to evaluate malaria control intervention impact [36], including bednets [19], case management [37, 38], and IRS [39]. The randomized design of MVIP was an opportune setting to evaluate the impact of the malaria vaccine with routine data [7]. Nevertheless, model outcomes may be adversely affected by data quality, data aggregation into age groups not aligned with vaccination ages, and site-specific factors.

Three key strategies were essential to overcome the limitations of routine surveillance data in this case. First, facilities consistently reporting malaria indicators, regardless of the overall reporting rate [17], were selected to reduce the impact of reporting bias. Five years of high-quality preevaluation data were required to develop the seasonal time-series models used to generate the 3-year counterfactual, yet this period experienced large changes in reporting. The large number of health facilities [40] and high vaccine uptake through the third dose [41] enabled an analysis restricted to only those facilities reporting 11 of 12 months each year for the entire study period. When the data were not restricted to consistently reporting facilities, no significant impact of RTS,S was detected when either the raw or the cleaned data (but not restricted to consistently reporting facilities) were used, likely because incomplete reporting, particularly during the pre-evaluation period when the models were created, led to inaccurate predictions. While most facilities did not report consistently, the included facilities represented 1.9 million reported malaria cases among children < 5 years. Restricting the analysis to consistently reporting facilities directly limited the role of reporting bias and coincidentally reduced the bias due to the unbalanced distribution of PBO-bednets during the evaluation period (2021). While the implementation of IRS remained imbalanced, it was more likely to have been accounted for in the time-series models because it occurred both before and during the evaluation period.

Second, this analysis required adjustment for contextual factors that may have influenced malaria case numbers unequally between implementing and comparison groups. In addition to an imbalance in vector control programs, there may have been imbalances in climate and rainfall [42], COVID-19 related reductions in health facility attendance [43], health worker strikes [44], and diagnostic and treatment supply disruptions [45]. Compared with an approach where each potential contextual factor was controlled for individually (e.g. separate covariates for bednet use and type, rainfall, etc.), ≥ 5y malaria cases, which were expected to have little or no impact from RTS,S, were included as a single covariate. This approach has been previously employed in other contexts with age restricted impacts [46], resulting in increased model validity, decreased prediction error, better adjustment for confounders, and narrower confidence intervals. With this approach, an impact on clinical malaria as small as ± 2.1% was detectable.

Finally, fitting multiple models and out-of-sample validation methods helped determine the best predictive model for the 3-year counterfactual. The wide range of SWAPE validation errors suggested that the choice of model could greatly influence the estimated impact. In this study, the ensemble models provided many of the best fitting models and one was chosen as the best fitting model for both implementing and comparison groups. An alternative model (‘optimized’, Fig. 7) estimated a greater impact but there was concern that this may have been because the models for each group were mathematically different rather than due to actual impact.

There are several limitations of the approach outlined here. The modeling methods may unintentionally bias the results. The data cleaning steps removed some values via algorithms that detect likely errors, but those errors were not validated against health facility source documentation. The selection of consistently reporting facilities may not be representative of all facilities, but imbalances in contextual factors and confounders have likely been controlled for by including ≥ 5yr cases in the predictive models. Additionally, using ≥ 5y cases as a covariate assumes that a reduction in malaria among vaccinated children would not reduce transmission intensity and thereby also reduce cases in older age groups. Should such indirect effects have occurred, the estimated impact here would underestimate the true population-level effect of the vaccine. Any analysis using this approach may face trade-offs between data quality and representativeness when attempting to measure vaccine impact.

To date, there has been only one study[47] in Ghana that attempted to estimate the impact of malaria vaccines via routine data. The Ghana study reported a reduction in clinical malaria incidence (− 33% [95%CI − 29%, − 36%] in 1–5 y.o.) more than three times greater than reported here and was greater than may have been expected from the vaccine coverage (75% for RTS,S dose 3 and 53% for dose 4 in Ghana [9]) and efficacy. Several mathematical modeling studies have produced predictions of the impact of malaria vaccines [48–52] on the basis of different assumptions, rather than comparisons of predicted and observed data, limiting comparability.

## Conclusions

For countries that implement malaria vaccines and are considering evaluating the impact with routine data, there are several important recommendations. First, ahead of the data analysis, estimate the expected impact given the endemicity of malaria, the expected vaccine coverage, and the age-range of cases reported to the routine surveillance system (HMIS). Second, delay impact evaluation until a substantial portion of the children within the HMIS reporting age-range are age-eligible to have received at least 3 doses. Third, critically evaluate the quality of data in the years preceding vaccine implementation to determine the number of consistently reporting facilities. Inconsistently reported data limit the ability to validate models and reduce the accuracy of predictions. Fourth, to control for confounding due to potentially contextual factors (e.g. vector-control interventions), especially when a suitable control is not possible, time-series models should include ≥ 5y cases as a covariate. Fifth, incorporating and validating multiple potential time-series models likely increases the accuracy and may avoid spurious results. Finally, the country should understand that a reduction in reported cases following introduction of the vaccine may not be causally linked to the vaccination. For example, declining cases with an associated vaccine impact could be the result of a vector control campaign or an interruption to malaria testing and treatment supplies. Similarly, there may be no apparent impact if other factors increase malaria transmission. RTS,S has been demonstrated to be efficacious however, attributing impact may not always be possible with routine data without both sufficient data quality and an appropriate control group.

## Disclaimer

MH, RO, and EF are affiliated to the World Health Organization. The authors alone are responsible for the views expressed in this publication, and they do not necessarily represent the views, decisions, or policies of the World Health Organization. AH, AS, EW, JP, and NW are affiliated to the US Centers for Disease Control and Prevention. The authors alone are responsible for the views expressed in this publication, and they do not necessarily represent the views, decisions, or policies of the US Centers for Disease Control and Prevention.

## Supplementary Information

Additional file1 (DOCX 1638 KB)

## Data Availability

The datasets generated and/or analysed during the current study are not publicly available owing to restrictions on access to routine health service data held within the Kenya Health Management Information System, in accordance with the data governance and confidentiality requirements of the Kenya Ministry of Health, but are available from the corresponding author upon reasonable request and subject to approval by the Kenya Ministry of Health. All the coding used to generate the results of this study is included in this published article and its supplementary information files.

## Abbreviations

< 5y: 0–59 Months
≥ 5y: 5 Years and older
ANT: Ensemble of the ARIMA (A), NNETAR (N), and TSLM (T) models
ARIMA: Autoregressive integrated moving average
ECDF: Empirical cumulative distribution function
ETS: Exponential smoothing
HMIS: Health management information system
IRS: Indoor residual spraying
KHIS: Kenya Health Management Information System
MOH: Ministry of Health
MVIP: Malaria Vaccine Implementation Programme
NNETAR: Neural network time-series forecast
OOS: Out-of-sample
PBO,: Piperonyl-butoxide-pyrethroid synergist-treated
RTS,S: RTS,S/AS01E
STL: Seasonal and trend decomposition via locally estimated scatterplot smoothing
SWAPE: Symmetric weighted absolute percentage error
TSLM: Time series linear model
WHO: World Health Organization
WPE: Weighted percentage error
