# Supplementary material for: The Malaria Vaccine Implementation Programme reduced clinical malaria in Kenya, 2020 to 2022: Impact evaluation with predictive time-series models of routine surveillance data
Source: Malar J. 2026 Jun 15;25:305. doi: 10.1186/s12936-026-05973-2 (PMC13501692; doi:10.1186/s12936-026-05973-2)
Supplement: Supplementary file 1 [file 12936_2026_5973_MOESM1_ESM.docx]

Supplemental material:

The Malaria Vaccine Implementation Programme Reduced Clinical Malaria in Kenya, 2020 to 2022

Impact evaluation with predictive time-series models of routine surveillance data

Table of contents

[**Supplement A: R Environment**	2](#_Toc226484068)

[**Supplement B: Expected impact**	4](#_Toc226484069)

[Potential Impact	6](#_Toc226484070)

[**Supplement C: Data Sources**	7](#_Toc226484071)

[Data Dictionary	7](#_Toc226484072)

[API Data Download	9](#_Toc226484073)

[**Supplement D: Enrolled Health Care Facilities**	10](#_Toc226484074)

[MVIP Areas	10](#_Toc226484075)

[MVIP Health Facilities	10](#_Toc226484076)

[**Supplement E: Vaccine Doses**	16](#_Toc226484077)

[**Supplement F: Confirmed Cases**	19](#_Toc226484078)

[Identification of anomalous values (outliers)	19](#_Toc226484079)

[Associate CHW cases with OPD cases	21](#_Toc226484080)

[**Supplement G: Consistently Reporting Facilities**	24](#_Toc226484081)

[Facilities Consistently Reporting Confirmed Cases Each Month	24](#_Toc226484082)

[**Supplement H: Time-series models of uncomplicated cases to estimate vaccine impact**	29](#_Toc226484083)

[Pre-implementation time-series models	29](#_Toc226484084)

[Validation	29](#_Toc226484085)

[Validation Algorithm: Optimizing versus Synchronizing	32](#_Toc226484086)

[Estimating Cumulative Impact: 1-12 Months Post-Implementation	34](#_Toc226484087)

[Estimating Cumulative Impact: 1-24 Months Post Implementation	37](#_Toc226484088)

[Estimating Cumulative Impact: 1-36 Months Post Implementation	39](#_Toc226484089)

[**Supplement I: Impact of short distance from comparison to implementing facilities**	41](#_Toc226484090)

[**Supplement J: Sensitivity Analysis**	44](#_Toc226484091)

[**References**	48](#_Toc226484092)

# **Supplement A: R Environment**

Data were prepared and analysed in RStudio 2024.4.0.735[1] running R version 4.4.1 with the following packages installed:

**Table S1.** R packages installed for analyses.

| Package | Version |
| --- | --- |
| coin | 1.4-3 |
| conflicted | 1.2.0 |
| cowplot | 1.2.0 |
| data.table | 1.17.8 |
| data.tree | 1.2.0 |
| distributional | 0.5.0 |
| DT | 0.34.0 |
| fable | 0.4.1 |
| fable.prophet | 0.1.0 |
| fabletools | 0.5.1 |
| feasts | 0.4.2 |
| fitdistrplus | 1.2-4 |
| flextable | 0.9.10 |
| forecast | 8.24.0 |
| fuzzyjoin | 0.1.6.1 |
| ggpattern | 1.2.1 |
| ggrepel | 0.9.6 |
| ggspatial | 1.1.10 |
| ggthemes | 5.2.0 |
| glue | 1.8.0 |
| gridExtra | 2.3 |
| gt | 1.1.0 |
| gtsummary | 2.4.0 |
| HDInterval | 0.2.4 |
| htmltools | 0.5.8.1 |
| igraph | 2.2.1 |
| kableExtra | 1.4.0 |
| knitr | 1.50 |
| leaflegend | 1.2.1 |
| leaflet | 2.2.3 |
| leaflet.extras | 2.0.1 |
| lubridate | 1.9.4 |
| lwgeom | 0.2-14 |
| magick | 2.9.0 |
| maps | 3.4.3 |
| MLmetrics | 1.1.3 |
| nngeo | 0.4.8 |
| overlapping | 2.2 |
| pacman | 0.5.1 |
| progress | 1.2.3 |
| readxl | 1.4.5 |
| scales | 1.4.0 |
| sf | 1.0-23 |
| stringr | 1.6.0 |
| table.express | 0.4.2 |
| tictoc | 1.2.1 |
| tidyfst | 1.8.2 |
| tidyverse | 2.0.0 |
| tsibble | 1.1.6 |
| webshot | 0.5.5 |
| webshot2 | 0.1.2 |

 R Code availability

The R code and all functions used in this analysis are available by request to the author, painter.ja@gmail.com

# **Supplement B: Expected impact**

The following inputs were used to calculate the expected reduction in uncomplicated malaria attributable to RTS,S, against which observed reductions were compared.

### Immunization Schedule

**Figure S1.** Immunization schedule, including RTS,S/AS01E In the three MVIP countries (Source: WHO MVIP SAP available at https://cdn.clinicaltrials.gov/large-docs/65/NCT03806465/SAP_002.pdf).

### Vaccine Eligibility

**Figure S2.** The proportion of all <5y children that were eligible for malaria vaccine started small and increased to 65% after 36 months post-introduction of RTS,S.

### Vaccine Coverage

From the western Kenya MVIP feasibility surveys, RTS,S coverage estimates among eligible children were 82.7% for dose 1, 78.1% for dose 2, 68.9% for dose 3, and 34.0% for dose 4. However, the eligible children were approximately 1/5 of all the children <5. Table S2 shows the estimated proportion of children <5 that received 3 or 4 doses following the introduction of RTS,S.

**Table S2.** Percent of under 5 population vaccinated by months since last dose

| Months since last dose | Year 1 | Year 2 | Year 3 |
| --- | --- | --- | --- |
| 6m post vx with 3 doses | 13.1% | 15.4% | 13.1% |
| 18m post vx with 3 doses | - | 7.2% | 7.2% |
| 30m post vx with 3 doses | - | - | 7.2% |
| 6m post vx with 4 doses | - | 5.9% | 7.2% |
| 18m post vx with 4 doses | - | - | 5.9% |

### Vaccine Efficacy

Vaccine efficacy decreased over time. Table S3 shows the vaccine efficacy for children receiving 3 and 4 doses of RTS,S/AS01_E._.[2,3] We assumed there was no efficacy unless children received at least 3 doses of RTS,S/AS01_E._

**Table S3**. Vaccine efficacy reported from phase III trials

| Months since last dose | 3 doses | 4 doses |
| --- | --- | --- |
| 6m post vx | 68.3% | 68.3% |
| 18m post vx | 45.1% | 45.1% |
| 30m post vx | 35.2% | 43.9% |
| 48m post vx | 28.3% | 36.0% |

## Potential Impact

The potential impact of the vaccine to reduce uncomplicated malaria cases was calculated as the product of the vaccine coverage and the vaccine efficacy.

**Table S4**. Potential impact of the vaccine to reduce uncomplicated malaria cases (coverage x efficacy)

| months | doses | Year 1 | Year 2 | Year 3 |
| --- | --- | --- | --- | --- |
| 6m post vx | 3 doses | 8.9% | 10.5% | 8.9% |
| 18m post vx | 3 doses | - | 3.2% | 3.2% |
| 30m post vx | 3 doses | - | - | 2.5% |
| 6m post vx | 4 doses | - | 4% | 4.9% |
| 18m post vx | 4 doses | - | - | 2.7% |
| Total | Total | 8.9% | 17.8% | 22.3% |

# **Supplement C: Data Sources**

 Note

Aggregated DHIS2 data are available through KHIS with access provided by Kenya Ministry of Health. Requests to access these datasets should be directed to Ministry of Health KHIS <https://hiskenya.org/dhis-web-commons/security/login.action> and KMHFL, [kmhfl@health.go.ke](http://kmhfl@health.go.ke/).

## Data Dictionary

We reviewed available data in the Kenya DHIS2 that represented monthly reporting of uncomplicated cases. Table S5 lists 5 monthly data elements with disaggregations that were included in the study dataset. Two of the three elements represent clinical cases reported by health facilities for persons <5 yrs and >=5 yrs. Three of the five represent clinical cases reported by community health education workers (CHEW). Prior to 2020, CHEW reported confirmed malaria without an age disaggregation. For this study, we categorized those cases as <5 so that they could be used to establish trends in <5 confirmed cases. In 2020, after expanding their role to testing and treating all ages KHIS enabled reporting clinical cases for <5 yrs and >=5 yrs.

KHIS also receives reports of uncomplicated malaria cases among pregnant women, which represents approximately 1% of uncomplicated malaria among persons >5 yrs. These data, however, were not available for the same period as the other uncomplicated cases, so we did not include them in our analyses.

**Table S5**. Dictionary of DHIS2 data elements accessed for confirmed cases

| dataElement | Categories | periodType | dataSet | zeroIsSignificant | data.id |
| --- | --- | --- | --- | --- | --- |
| CHEW No. positive | default | Monthly | MOH 748 - COMMUNITY HEALTH UNIT MONTHLY SUMMARY REPORT FOR MALARIA COMMODITIES | FALSE | CVHsV294PjK.NhSoXUMPK2K |
| CHEW No. positive < 5 | default | Monthly | MOH 748 - COMMUNITY HEALTH UNIT MONTHLY SUMMARY REPORT FOR MALARIA COMMODITIES | FALSE | NeUMw0K561Q.NhSoXUMPK2K |
| CHEW No. positive >= 5 | default | Monthly | MOH 748 - COMMUNITY HEALTH UNIT MONTHLY SUMMARY REPORT FOR MALARIA COMMODITIES | FALSE | TZHFXJrxbUU.NhSoXUMPK2K |
| Confirmed Malaria (only Positive cases) | >5 yrs | Monthly | AWP Monthly Service Delivery;Family Health, PVLS, malaria & Consolidated Dashboards - USAID 2022;HIS-PMS DATA EXCHANGE;MOH 705 A Outpatient summary < 5 years Revised 2020;MOH 705 B Outpatient summary > 5 years Revised 2020 | TRUE | OoakJhWiyZpeDaCnHi3m5 |
| Confirmed Malaria (only Positive cases) | <5 yrs | Monthly | AWP Monthly Service Delivery;Family Health, PVLS, malaria & Consolidated Dashboards - USAID 2022;HIS-PMS DATA EXCHANGE;MOH 705 A Outpatient summary < 5 years Revised 2020;MOH 705 B Outpatient summary > 5 years Revised 2020 | TRUE | OoakJhWiyZp.lHl2rmXpHse |

The “data.id” column was created by concatenating the dataElement id and the categoryOptionCombo id with “.” separator.

 **Common terms used with DHIS2 reporting**

**Data Element**: A data element represents the smallest unit of data that can be collected and analyzed in DHIS2. It could be a single piece of information such as the number of patients, the prevalence of a disease, or any other relevant health-related indicator. Each data element typically has a name, code, description, and data type associated with it.

**Categories**: Categories are used to define dissagregations like age group, and gender.

**Dataset**: A dataset in DHIS2 is a collection of related data elements that are grouped together for data collection, analysis, and reporting purposes. Datasets are typically used to organize data collection forms or data entry screens.

**ZeroIsSignificant:** a boolean property in DHIS2 data elements (true/false) that dictates whether an entered zero (0) is stored as a significant value or treated as null/empty. When true, DHIS2 stores the zero value; when false (default), zeros are ignored in data entry and treated as null, impacting completeness and validation.

## API Data Download

The data.id in Table S6 were downloaded from KHIS (https://hiskenya.org, DHIS2 version 2.35.5) using the DHIS2 API[4] to request data for all organizational units (orgUnits) each month from January 2015 through December 2022 (data accessed on 2024-09-20). Data were first downloaded with the option “SUM”; then with the option “COUNT”; and combined into one R dataset. For illustration, a small sample of the data (5 of 1,444,124 rows) is shown in Table S6.

**Table S6**. Sample of data downloaded for confirmed cases

| dataElement | categoryOptionCombo | period | orgUnit | COUNT | SUM |
| --- | --- | --- | --- | --- | --- |
| OoakJhWiyZp | SeDaCnHi3m5 | 201301 | A2FJEDRHsgv | 1 | 1 |
| OoakJhWiyZp | SeDaCnHi3m5 | 201303 | A2FJEDRHsgv | 1 | 30 |
| OoakJhWiyZp | SeDaCnHi3m5 | 201306 | A2FJEDRHsgv | 1 | 3 |
| OoakJhWiyZp | SeDaCnHi3m5 | 201307 | A2FJEDRHsgv | 1 | 1 |
| OoakJhWiyZp | SeDaCnHi3m5 | 201309 | A2FJEDRHsgv | 1 | 11 |

The value of SUM is the total value (number of confirmed cases) from the orgUnit on that row, including the total value from any subordinate facilities. An orgUnit may represent a health provider (e.g. community health worker or health facility) as well as an administrative unit (e.g. sub-district, district, region, etc.). The value of COUNT represents the number of facilities reporting the variable (data.id). When COUNT >1, then the value includes totals from subordinate orgUnits, for example a sub-district with multiple health facilities reporting to it. The number of subordinate orgUnits that report the specified variable (data.id) each month frequently varies. For orgUnits that do not have any subordinates, e.g. community health workers, the COUNT will always be 1.

# **Supplement D: Enrolled Health Care Facilities**

## MVIP Areas

**Table S7**. Health units associated with each organization unit level

| Intervention | County | Sub Counties |
| --- | --- | --- |
| Comparison | Bungoma | Kanduyi, Sirisia |
| Comparison | Busia | Bunyala, Matayos, Teso North, Teso South |
| Comparison | Homa Bay | Karachuonyo, Rachuonyo South, Mbita, Rangwe, Suba South |
| Comparison | Kakamega | Lurambi, Matungu, Mumias East, Shinyalu |
| Comparison | Kisumu | Nyakach |
| Comparison | Migori | Rongo, Suna East, Suna West, Uriri |
| Comparison | Siaya | Ugunja |
| Comparison | Vihiga | Hamisi, Sabatia |
| Implementing | Bungoma | Bumula |
| Implementing | Busia | Butula, Samia, Nambale |
| Implementing | Homa Bay | Homa Bay Town, Kabondo Kasipul, Ndhiwa |
| Implementing | Kakamega | Butere, Ikolomani, Khwisero, Malava, Mumias West, Navakholo |
| Implementing | Kisumu | Kisumu Central, Kisumu East, Muhoroni |
| Implementing | Migori | Awendo, Nyatike |
| Implementing | Siaya | Rarieda, Ugenya |
| Implementing | Vihiga | Emuhaya, Luanda, Vihiga |

## MVIP Health Facilities

A list of organizational units, which includes health facilities was downloaded (Sep 9, 2023) from KHIS using the DHIS2 API[4], for each organisational unit (orgUnit) level (1-6) (Table S8 ):

API for all orgUnits in a level: https://hiskenya.org/api/organisationUnits.geojson?level=2&fields=:all&paging=false

**Table S8**. Example of orgUnits available from a DHIS2 instance (showing 5 of 28,359 rows)

| name | orgUnit | level | levelName | parentOrgUnit | leaf |
| --- | --- | --- | --- | --- | --- |
| Baringo County | vvOK1BxTbet | 2 | County | Kenya | FALSE |
| Bomet County | HMNARUV2CW4 | 2 | County | Kenya | FALSE |
| Bungoma County | KGHhQ5GLd4k | 2 | County | Kenya | FALSE |
| Baringo Central Sub County | k7Rj54u6dMx | 3 | Sub-County | Baringo County | FALSE |
| Baringo North Sub County | bqtTmWcikTN | 3 | Sub-County | Baringo County | FALSE |
| East Pokot Sub County | Mk4bMOSMRTB | 3 | Sub-County | Baringo County | FALSE |
| Ainabkoi/Olare Ward | keTxMrb6Oag | 4 | County Assembly Ward | Ainabkoi Sub County | FALSE |
| Kapsoya Ward | H9e1tEercdW | 4 | County Assembly Ward | Ainabkoi Sub County | FALSE |
| Kaptagat Ward | cYwuj1W0Wux | 4 | County Assembly Ward | Ainabkoi Sub County | FALSE |
| Abakaile Dispensary | QITW06phVzk | 5 | Health Facility | Abakaile Ward | FALSE |
| Alikune Dispensary | edjfwbsoTLn | 5 | Health Facility | Abakaile Ward | FALSE |
| Kumahumato Dispensary | J2nSU1CrG7s | 5 | Health Facility | Abakaile Ward | FALSE |
| Ayucha Community Health Unit | lsAviUAjWGL | 6 | CommunityUnit | Ayucha Heallth Centre | TRUE |
| Checkpoint Community Unit | TlYwJC9qpNb | 6 | CommunityUnit | Nakuru West( A.I.P.C.A) Medical Centre | TRUE |
| Honeycup Community Health Unit | JsZeP9JbXsS | 6 | CommunityUnit | Nakuru West( A.I.P.C.A) Medical Centre | TRUE |

 **Organisational Hierarchy in DHIS2**

“The [orgUnit] hierarchy is built up of parent-child relations, e.g. a Country or MoH unit (the root) might have e.g. 8 parent units (provinces), and each province again (at level 2) might have 10-15 districts as their children. Normally the health facilities will be located at the lowest level, but they can also be located at higher levels, e.g. national or provincial hospitals, so skewed organisational trees are supported (e.g. a leaf node can be positioned at level 2 while most other leaf nodes are at level 5).” (DHIS2 Documentation[5])

Note: ‘leaf’ is a logical variable in DHIS2 that refers to the lowest level of a tree-like structure. When TRUE, there is no orgUnit below. When FALSE, there is at least one orgUnit below whose data will be aggregated into the totals for this orgUnit (see DHIS2 Documentation in the box below).

The KHIS organization units are organized by levels (Table S9).

**Table S9**. Organisational Unit Levels in KHIS

| level | levelName |
| --- | --- |
| 1 | Kenya |
| 2 | County |
| 3 | Sub-County |
| 4 | County Assembly Ward |
| 5 | Health Facility |
| 6 | CommunityUnit |

Note: The orgUnit levels in DHIS2 are different from the Kenya Public Health service levels, which are organized by level of care [6].

**Table S10.** Number of orgUnits associated with each KHIS level

| level | levelName | Facility Key Word | No. of Units |
| --- | --- | --- | --- |
| 4 | County Assembly Ward | Hospital | 3 |
| 5 | Health Facility | Clinic | 3,392 |
| 5 | Health Facility | Community | 254 |
| 5 | Health Facility | Health | 2,078 |
| 5 | Health Facility | Hospital | 1,038 |
| 6 | CommunityUnit | Clinic | 7 |
| 6 | CommunityUnit | Community | 9,447 |
| 6 | CommunityUnit | Health | 247 |
| 6 | CommunityUnit | Hospital | 11 |

### MVIP-KHIS Health Facilities

An additional facility list for the MVIP area was obtained with additional information about the facilities, including the ownership (public vs. private), KEPH service level, and updated coordinates.[7]

DHISID was ‘None’ for 109 orgUnits. We attempted to match the Facility_Name with the orgUnit name in KHIS. The DHISID was added for 6 of 109 rows.

### Facility types in MVIP areas

**Table S11**. Type of health units in MVIP districts associatied with each organization unit level

| KHIS Level | Comparison | Implementing |
| --- | --- | --- |
| Sub-County | 23 | 23 |
| County Assembly Ward | 115 | 113 |
| Health Facility | 874 | 840 |
| CommunityUnit | 838 | 931 |

**Table S12**. Type of health units in MVIP districts associated with each organization unit level

| KHIS Level | Owner | Comparison | Implementing |
| --- | --- | --- | --- |
| Health Facility | Ministry of Health | 392 | 399 |
| Health Facility | Mission/Faith-Based Organization | 80 | 65 |
| Health Facility | Non-Governmental Organization | 16 | 29 |
| Health Facility | Other Min | 14 | 9 |
| Health Facility | Private | 255 | 234 |
| Health Facility | Unidentified | 117 | 104 |

### Percentage of facility ownership by intervention

For facilities in KHIS that were linked with the master list, we compared the facility ownership across interventions Table S13. (The column with owner, the row with missing value for owner indicates facilities in the master list that were not linked with those in KHIS)

**Table S13**. Ownership of health units for the MVIP intervention areas

| Owner | Comparison | Implementing |
| --- | --- | --- |
| Ministry of Health | 38.7% | 40.9% |
| Mission/Faith-Based Organization | 7.9% | 6.7% |
| Non-Governmental Organization | 1.6% | 3.0% |
| Other MOH | 1.4% | 0.9% |
| Private | 25.2% | 24.0% |
| Unknonwn | 25.2% | 24.6% |

### Vector Control Strategy Among Intervention Areas

Sub-counties in Western Kenya participated in several vector control strategies. IRS was applied annually in 14 sub-counties. Meanwhile, mass bednet campaigns were carried out in 32 sub-counties during 2021. Of these, 12 sub-counties received long-lasting insecticide nets treated with pyrethroids (Standard net) and 20 received long-lasting insecticide nets treated with combined pyrethroid and piperonyl-butoxide (PBO).[8] A greater proportion of sub-district assigned to the comparison groups received the more effective vector control interventions, IRS and PBO nets (Table S14).

**Table S14**. Vector control interventions assigned to sub-counties in each MVIP intervention area

| Intervention | Vector Control (2021) | No.  Counties | No.  Sub Counties | No.  Facilities | Percent |
| --- | --- | --- | --- | --- | --- |
| Comparison | IRS | 2 | 9 | 383 | 44.0% |
| Comparison | PBO | 3 | 10 | 344 | 39.5% |
| Comparison | Pyrethroid | 3 | 4 | 144 | 16.5% |
| Implementing | IRS | 2 | 5 | 244 | 29.2% |
| Implementing | PBO | 3 | 10 | 270 | 32.3% |
| Implementing | Pyrethroid | 3 | 8 | 322 | 38.5% |
|  | Total | 16 | 46 | 1,707 | 200.0% |

# **Supplement E: Vaccine Doses**

We downloaded monthly malaria vaccine data by facility reported through DHIS2 from 2018 to 2022 on 2023-08-26.

**Table S15**. Number of monthly malaria vaccine reports by dose

| data.id | data | vx_reports |
| --- | --- | --- |
| Rhkz9D6hdZV_NhSoXUMPK2K | NVIP Malaria Vaccine Dose 2_default | 27020 |
| U46JIUFqgvj_NhSoXUMPK2K | NVIP Malaria Vaccine Dose 1_default | 27906 |
| pznTlBTeIb7_NhSoXUMPK2K | NVIP Malaria Vaccine Dose 4_default | 17266 |
| znLJPgpYjpF_NhSoXUMPK2K | NVIP Malaria Vaccine Dose 3_default | 25656 |

**Table S16**. Number of subcounties providing RTS,S reports

| Intervention | vx_reports |
| --- | --- |
| Comparison | 23 |
| Implementing | 23 |
| non-MVIP | 16 |

Several additional sub-counties provided vaccination during the MVIP

**Table S17**. Non-MVIP vaccination sub-counties

| County | Sub-County |
| --- | --- |
| Kakamega County | Mumias West Sub County |
| Kisumu County | Kisumu Central Sub County |
| Kisumu County | Kisumu East Sub County |
| Kisumu County | Kisumu West Sub County |
| Kisumu County | Seme Sub County |
| Siaya County | Gem Sub County |

**Table S18.** Complete list of MVIP and non-MVIP subcounties

| Intervention | County | Sub-County | Vaccine Reports |
| --- | --- | --- | --- |
| Comparison | Bungoma County | Kanduyi Sub County | 399 |
| Comparison | Bungoma County | Sirisia Sub County | 224 |
| Comparison | Busia County | Bunyala Sub County | 134 |
| Comparison | Busia County | Matayos Sub County | 256 |
| Comparison | Busia County | Teso North Sub County | 130 |
| Comparison | Busia County | Teso South Sub County | 260 |
| Comparison | Homa Bay County | Karachuonyo Sub County | 657 |
| Comparison | Homa Bay County | Mbita Sub County | 369 |
| Comparison | Homa Bay County | Rachuonyo South Sub County | 335 |
| Comparison | Homa Bay County | Rangwe Sub County | 335 |
| Comparison | Homa Bay County | Suba South Sub County | 331 |
| Comparison | Kakamega County | Lurambi Sub County | 319 |
| Comparison | Kakamega County | Matungu Sub County | 249 |
| Comparison | Kakamega County | Mumias East Sub County | 210 |
| Comparison | Kakamega County | Shinyalu Sub County | 317 |
| Comparison | Kisumu County | Nyakach Sub County | 373 |
| Comparison | Migori County | Rongo Sub County | 220 |
| Comparison | Migori County | Suna East Sub County | 191 |
| Comparison | Migori County | Suna West Sub County | 283 |
| Comparison | Migori County | Uriri Sub County | 315 |
| Comparison | Siaya County | Ugunja Sub County | 212 |
| Comparison | Vihiga County | Hamisi Sub County | 459 |
| Comparison | Vihiga County | Sabatia Sub County | 181 |
| Implementing | Bungoma County | Bumula Sub County | 2790 |
| Implementing | Busia County | Butula Sub County | 2842 |
| Implementing | Busia County | Nambale Sub County | 2130 |
| Implementing | Busia County | Samia Sub County | 2362 |
| Implementing | Homa Bay County | Homa Bay Town Sub County | 3091 |
| Implementing | Homa Bay County | Kabondo Kasipul Sub County | 3748 |
| Implementing | Homa Bay County | Ndhiwa Sub County | 5909 |
| Implementing | Kakamega County | Butere Sub County | 2770 |
| Implementing | Kakamega County | Ikolomani Sub County | 2740 |
| Implementing | Kakamega County | Khwisero Sub County | 2902 |
| Implementing | Kakamega County | Malava Sub County | 4395 |
| Implementing | Kakamega County | Mumias West Sub County | 2127 |
| Implementing | Kakamega County | Navakholo Sub County | 2408 |
| Implementing | Kisumu County | Kisumu Central Sub County | 4438 |
| Implementing | Kisumu County | Kisumu East Sub County | 3700 |
| Implementing | Kisumu County | Muhoroni Sub County | 4363 |
| Implementing | Migori County | Awendo Sub County | 3252 |
| Implementing | Migori County | Nyatike Sub County | 6773 |
| Implementing | Siaya County | Rarieda Sub County | 4306 |
| Implementing | Siaya County | Ugenya Sub County | 2917 |
| Implementing | Vihiga County | Emuhaya Sub County | 1760 |
| Implementing | Vihiga County | Luanda Sub County | 2810 |
| Implementing | Vihiga County | Vihiga Sub County | 2368 |
| non-MVIP | Busia County | Bunyala Sub County | 23 |
| non-MVIP | Busia County | Matayos Sub County | 16 |
| non-MVIP | Busia County | Teso North Sub County | 11 |
| non-MVIP | Busia County | Teso South Sub County | 2 |
| non-MVIP | Homa Bay County | Mbita Sub County | 2 |
| non-MVIP | Kakamega County | Lurambi Sub County | 20 |
| non-MVIP | Kakamega County | Mumias West Sub County | 126 |
| non-MVIP | Kisumu County | Kisumu Central Sub County | 167 |
| non-MVIP | Kisumu County | Kisumu East Sub County | 316 |
| non-MVIP | Kisumu County | Kisumu West Sub County | 3847 |
| non-MVIP | Kisumu County | Nyando Sub County | 340 |
| non-MVIP | Kisumu County | Seme Sub County | 3587 |
| non-MVIP | Migori County | Suna East Sub County | 3 |
| non-MVIP | Siaya County | Bondo Sub County | 375 |
| non-MVIP | Siaya County | Gem Sub County | 5321 |
| non-MVIP | Vihiga County | Sabatia Sub County | 32 |

- Before 2023, only facilities in the implementing areas reported administering RTS,S doses 1-4. Facilities in the Comparison areas began implementing in 2023.
- Approximately 75 facilities in sub-districts adjacent to the MVIP reporting administering RTS,S doses 1-4 during 2020-2022.

# **Supplement F: Confirmed Cases**

Data were downloaded from KHIS through the DHIS2 API. Successive API calls were made for each data element for all months from Jan 2013 through Dec 2022. An example of the format of the downloaded data was shown in Supplement C.

**Table S19**. Sample of data downloaded for confirmed cases

| dataElement | categoryOptionCombo | period | orgUnit | COUNT | SUM |
| --- | --- | --- | --- | --- | --- |
| OoakJhWiyZp | SeDaCnHi3m5 | 201301 | A2FJEDRHsgv | 1 | 1 |
| OoakJhWiyZp | SeDaCnHi3m5 | 201303 | A2FJEDRHsgv | 1 | 30 |
| OoakJhWiyZp | SeDaCnHi3m5 | 201306 | A2FJEDRHsgv | 1 | 3 |
| OoakJhWiyZp | SeDaCnHi3m5 | 201307 | A2FJEDRHsgv | 1 | 1 |
| OoakJhWiyZp | SeDaCnHi3m5 | 201309 | A2FJEDRHsgv | 1 | 11 |

The raw data file was downloaded from KHIS on 2024-09-20 and was prepared by mapping the data element codes to their corresponding names and converting the R data frame to a tsibble[10] time-series dataset using the data functions available at <https://github.com/jpainter/MG2>.

The reported clinical cases were linked with the MVIP health facilities and restricted to data submitted prior to January 2023 with the following conditions:

- Restricted to facilities in MVIP that are below KHIS level 4, i.e. only primary care facilities
- Created agegrp variable for cases <5y and >5y.
- Confirmed that all the confirmed cases were MVIP providers.

## Identification of anomalous values (outliers)

Anomalous values were identified using a sequential set of algorithms, each applied to the residuals remaining after the previous step. Algorithms were applied independently to each data series defined by the combination of organisational unit, data element, and age category.

### Step 1: Global outlier detection using median absolute deviation (MAD)

The first two algorithms identified values that were extreme relative to the overall distribution of the series, using the median absolute deviation (MAD) — a robust measure of variability analogous to the standard deviation but less sensitive to the influence of multiple outliers (Leys et al., 2013). For each series, we calculated:

outlier = |( x̂ − x ) / MAD| ≥ deviation

where x is the observed value, x̂ is the expected value from the cleaned series (see below), and deviation is the threshold. We applied this first with a threshold of 15 (MAD15) and then applied the same algorithm to the remaining values with a threshold of 10 (MAD10), allowing detection of both extreme and moderately extreme global outliers sequentially.

### Step 2: Seasonal outlier detection

After removing extreme global outliers, we applied two further algorithms to identify values that were anomalous relative to the expected seasonal pattern — that is, values that may fall within the overall range of the series, but whose magnitude is inconsistent with observations from the same time of year in other years.

Expected values were generated using the tsclean function from the R forecast package (Hyndman & Khandakar, 2008), which interpolates missing values and smooths the series while preserving its seasonal structure. The MAD was used to standardize the deviation from expectation.

The seasonal5 algorithm flagged values where the standardized deviation from expectation was five-fold or greater:

outlier = |( x̂ − x ) / MAD| ≥ 5

Values passing this threshold were then re-evaluated using the seasonal3 algorithm, which applied a stricter threshold of three-fold:

outlier = |( x̂ − x ) / MAD| ≥ 3

Applying these algorithms sequentially (seasonal5 first, then seasonal3 on the remaining values) allowed progressively finer identification of values whose timing was incongruous with seasonal norms, without over-flagging moderate seasonal variation.

### Implementation

All algorithms were implemented in R. The core logic for each step is as follows, where x is the series of data values:

x.ts <- x %>% as.ts(., frequency = 12)

x.forecast <- tsclean( x.ts, replace.missing = "interpolate", lambda = 0.5 ) %>%

as.integer()

MAD <- mad(x, na.rm = TRUE)

outlier <- abs((x.forecast - x.ts) / MAD) >= deviation

The R functions and code may be found in the R package, MG2, available on GitHub (<https://github.com/jpainter/MG2>).

**Table S20.** Number of errors flagged with the outlier algorithms

| Error Flag | N | %N | Total Value | % TotalValue |
| --- | --- | --- | --- | --- |
| mad15 | 219 | 0.1% | 111,509 | 0.5% |
| mad10 | 371 | 0.1% | 116,890 | 0.5% |
| seasonal5 | 691 | 0.3% | 289,267 | 1.2% |
| seasonal3 | 1,548 | 0.6% | 509,776 | 2.2% |
| No Errors | 257,517 | 99.1% | 509,776 | 96.5% |

## Associate CHW cases with OPD cases

Community cases were associated with health facilities and should be combined before scanning for consistently reporting facilities. We assume that community cases occurred in the catchment area of their parent facility and that to assess the trends in cases in each catchment area, the community cases should be combined with their respective health facility cases. We assume that cases diagnosed at community would otherwise have been diagnosed at the health facility. However, it is possible that the increased access to care provided by CHW (starting Jan 2018) could have increased the total number of reported cases.

Note that CHW replaced the default category with “<5” and “>5” in 2021. Unfortunately, the community cases in 2018-late 2021 were not age disaggregated and therefore cannot be included in age-specific models of vaccine impact. We included the cases reported with age disaggregation on the revised reporting form in late 2021.

We combined the CHEW cases with their respective health facility with a new variable to indicate age group and source of confirmation (CHEW vs OPD) data from CHEW and facilities

When age-groups reporting was available for CHEW (2021-2022), under5 accounted for 35.4% of cases. Applying that percentage to the unknown age CHEW cases suggests 333,124 under5 cases from 2018-2020 may not be included. This was approximately 20.3% of OPD cases during same years.

**Figure S3.** Number of confirmed cases reported by CHW or OPD by age group.

### Associate CHW cases with their corresponding OPD, creating a new variable, opdUnit

Some CHEW report using the same orgUnit id as the health facilities that report OPD cases; some report using the health facility as their parent; and some CHEW report to the health facility two levels above (e.g. grandparent). Cases reported by CHW are possibly cases that would otherwise have been reported by the OPD. Therefore, we associate all these CHEW reports to their respective OPD orgUnit (‘effectiveOrgUnit’) before aggregating the data. The data for each age group was aggregated to each health facility. We used that data to determine facilities that consistently report. We associated 519 of 521 CHEW orgUnits with their parent OPD.

CHW case data were associated with their OPD and summarized across each OrgUnit by Intervention and age group.

# **Supplement G: Consistently Reporting Facilities**

**Figure** **S4**. Mean number of OPD facilities reporting confirmed cases each year by intervention area

## Facilities Consistently Reporting Confirmed Cases Each Month

To assess the potential reporting bias, we examined the consistency of monthly reporting. Ideally, all facilities report each and every month.

To remove reporting bias from the evaluation period, we decided to include only those facilities that reported each and every month during the study period (Jan 2020 - Dec 2022) and as many months as possible prior to Jan 2020. In general, at least 24 months of data is required to develop prediction models with seasonal data, and an additional 12 months of data is required to validate the prediction models. Therefore, we assessed the number of consistently reporting facilities from no later than Jan 2017 through Dec 2022.

As facilities were being added to KHIS-DHIS2, more facilities reported each year up to and including 2017. Therefore, there will likely be a tradeoff between having more months available for modeling and the number of facilities consistently reporting.

A facility was counted as having reported consistently if either the facility or associated CHW reported for that month. In some months the CHW did not report, so this assumes that when CHW did not report cases, the cases were reported by the facility. This could be the case when CHW are stocked out of test kits and patients could only be tested at the nearest facility.

**Table S23.** Number of Facilities Reporting Confirmed Cases Each Month

allowing 0-2 missing reports per year. The criteria selected for the final analysis are highlighted in bold.

|  |  | No. Missing report / year | | |
| --- | --- | --- | --- | --- |
|  | Starting | 0 | 1 | 2 |
| Comparison | 2013 Jan | 43 | 108 | 148 |
| Implementing | 2013 Jan | 28 | 97 | 144 |
| Comparison | 2013 Jul | 46 | 118 | 156 |
| Implementing | 2013 Jul | 31 | 106 | 152 |
| Comparison | 2014 Jan | 48 | 120 | 162 |
| Implementing | 2014 Jan | 35 | 108 | 155 |
| Comparison | 2014 Jul | 52 | 134 | 180 |
| Implementing | 2014 Jul | 42 | 124 | 177 |
| **Comparison** | **2015 Jan** | 55 | **139** | 182 |
| **Implementing** | **2015 Jan** | 47 | **132** | 182 |
| Comparison | 2015 Jul | 61 | 147 | 190 |
| Implementing | 2015 Jul | 51 | 140 | 191 |
| Comparison | 2016 Jan | 66 | 151 | 194 |
| Implementing | 2016 Jan | 59 | 146 | 197 |
| Comparison | 2016 Jul | 72 | 160 | 204 |
| Implementing | 2016 Jul | 68 | 158 | 212 |
| Comparison | 2017 Jan | 81 | 165 | 210 |
| Implementing | 2017 Jan | 72 | 165 | 216 |
| Comparison | 2017 Jul | 85 | 173 | 227 |
| Implementing | 2017 Jul | 83 | 180 | 241 |
| Comparison | 2018 Jan | 118 | 206 | 268 |
| Implementing | 2018 Jan | 112 | 235 | 314 |
| Comparison | 2018 Jul | 126 | 219 | 281 |
| Implementing | 2018 Jul | 127 | 249 | 327 |
| Comparison | 2019 Jan | 147 | 243 | 307 |
| Implementing | 2019 Jan | 137 | 261 | 339 |
| Comparison | 2019 Jul | 163 | 263 | 336 |
| Implementing | 2019 Jul | 156 | 277 | 357 |
| Comparison | 2020 Jan | 198 | 330 | 405 |
| Implementing | 2020 Jan | 206 | 348 | 419 |
| Comparison | 2020 Jul | 237 | 385 | 468 |
| Implementing | 2020 Jul | 240 | 396 | 476 |

To select the parameters for consistently reporting facilities we decided on the following criteria:

1. To have the fewest number of missing months-ideally zero-as any missed month introduces unknown bias.
2. To have at least 3 years of data prior to the intervention to have sufficient data for validating the models used to predict the number of cases during the intervention period (the counterfactual).
3. To include as many facilities as possible, with a minimum of at least 100 facilities in each intervention to make a representative sample.

Thus, we chose a starting month of Jan 2015 and allowed up to one missing month of data per year. This yielded 139 comparison facilities and 132 implementing facilities. Figure S5 shows location of the facilities and Table S24 provides selected characteristics.

**Table S24**. Characteristics of facilities included (Selected = TRUE) versus excluded (Selected = FALSE).

| **Intervention** | **Selected** | **Facilities** | **MOH^a^** | **<5y Malaria**  **(%)** | **RTS,S Doses**  **(%)** | **PBO-nets^b^** | **IRS^c^** |
| --- | --- | --- | --- | --- | --- | --- | --- |
| Comparison | TRUE | 139 | 60.4% | 1,004,891  (18.6%) | 0  (0.0%) | 46.8% | 33.8% |
| Implementing | TRUE | 132 | 59.1% | 944,082  (17.5%) | 374,001  (40.3%) | 45.5% | 18.9% |
| Comparison | FALSE | 642 | 55.0% | 1,594,685  (29.6%) | 0  (0.0%) | 43.3% | 38.8% |
| Implementing | FALSE | 615 | 56.9% | 1,852,058  (34.3%) | 554,645  (59.7%) | 32.4% | 24.5% |
| a: Percent of facilities owned by Ministry of Health. Other facilities owned by 'Private', 'Mission/Faith-Based Organization', or 'Non-Governmental Organization'. Owner was not indicated for 24.8% of facilities | | | | | | | |
| b: Percent of facilities where PBO nets distributed during mass campaign in 2021 | | | | | | | |
| c: Percent of facilities where IRS applied annually | | | | | | | |

**Figure S5**. Map of facilities included in final analytic dataset

# **Supplement H: Time-series models of uncomplicated cases to estimate vaccine impact**

## Pre-implementation time-series models

We developed time-series model[14], including all permutations[15] of the ARIMA, ETS, NNETAR, and prophet models. Predictions were then generated for the last 12 months of the pre-intervention period. We compared those predictions with the actual values to validate the models.

Some key model details:

- The exogenous variable, older, was log-transformed for the ARIMA model because models with the untransformed variable warned of instability. None of the other models produced a warning and the results showed improved model fit (slightly smaller SWAPE) when untransformed.
- Parameters for each primary model were selected with each package’s auto-fitting method. The reported number over-5 malaria cases were included as a covariate in each model except ETS, which does not allow covariates. Values for the dependent variable, under-5 cases, were log-transformed to stabilize variance and ensure that predicted values were non-negative. For ARIMA models, the exogenous regressor was also log-transformed to prevent undefined predicted values. No undefined values were generated by other models and the model fit was improved with an untransformed regressor.
- For prophet model[22], the seasonal component has two parameters:
  - order: The number of terms in the partial Fourier sum. The higher the order, the more flexible the seasonality can be. (1 is least - more prone to under fitting, and 8 is most - more likely to overfit )
  - type: The nature of the seasonality. If “additive”, the variability in the seasonal pattern is fixed. If “multiplicative”, the seasonal pattern varies proportionally to the level of the series. We used ‘multiplicative’.

## Validation

To test the validity of each model we compared the predicted values with the actual values during the calendar year 2019. The predicted values are generated[16] from each model using the known values for confirmed cases in persons 5 and older during the calendar year 2019.

To assess the model’s accuracy for forecasting, it is important to use a metric that evaluates the accuracy of a forecast. A general process for validating models, commonly used in machine learning, involves fitting a number of models with different characteristics to a training dataset and then testing them on a validation set.[17] If the accuracy were to have been determined based on the goodness of fit with the training data, it is very likely the data will be overfit and not do well when forecasting values not in the training set. Therefore, we reserved data from the training set that can be used to see how well the model predicts the missing values.

To assess the models forecast accuracy, we used a metric that evaluates the accuracy of a forecast. Metrics like the mean absolute percent error (MAPE) [18] penalizes the forecast for error above and below the actual value. The MAPE has limitations[19,20] however, and many other metrics have been proposed.[17]

For in-sample validation, the RMSE was chosen to validate the models because the model fitting functions themselves (e.g. ARIMA) use the RMSE to optimize their parameters, and therefore we also used RMSE to select the best fit.[21]

### 1. In-sample validation performance

**Table S25**. In-sample validation performance

| Intervention | Model | Type | ME | RMSE | MAE | MPE | MAPE | MASE | RMSSE | ACF1 | rank |
| --- | --- | --- | --- | --- | --- | --- | --- | --- | --- | --- | --- |
| Comparison | p8 | Training | 2.3200 | 719 | 554 | -0.2060 | 4.90 | 0.2170 | 0.2190 | 0.2450 | 1 |
| Comparison | a | Training | -35.6000 | 800 | 643 | -0.2280 | 5.90 | 0.2520 | 0.2430 | -0.0205 | 2 |
| Comparison | n | Training | 40.8000 | 826 | 673 | -0.3020 | 6.23 | 0.2640 | 0.2510 | 0.3510 | 3 |
| Comparison | p4 | Training | 5.3400 | 867 | 686 | -0.3000 | 6.11 | 0.2690 | 0.2640 | -0.0549 | 4 |
| Comparison | p1 | Training | 6.2000 | 877 | 697 | -0.3270 | 6.25 | 0.2730 | 0.2670 | 0.0166 | 5 |
| Comparison | t | Training | 11.7000 | 941 | 733 | -0.3740 | 6.52 | 0.2870 | 0.2870 | 0.2570 | 6 |
| Comparison | e | Training | -48.4000 | 1,500 | 1,130 | -1.5100 | 10.00 | 0.4450 | 0.4550 | 0.1070 | 7 |
| Implementing | n | Training | 19.6000 | 320 | 216 | -0.0047 | 1.87 | 0.0818 | 0.0953 | -0.0452 | 1 |
| Implementing | p8 | Training | -3.7500 | 968 | 648 | -0.2800 | 5.64 | 0.2450 | 0.2880 | 0.4280 | 2 |
| Implementing | a | Training | -70.9000 | 914 | 651 | -0.5690 | 5.64 | 0.2460 | 0.2720 | 0.1340 | 3 |
| Implementing | t | Training | 0.0973 | 1,060 | 739 | -0.3930 | 6.71 | 0.2800 | 0.3140 | 0.3000 | 4 |
| Implementing | p4 | Training | -0.3940 | 1,030 | 759 | -0.3440 | 6.67 | 0.2870 | 0.3080 | 0.2250 | 5 |
| Implementing | p1 | Training | 3.6500 | 1,140 | 806 | -0.4040 | 7.13 | 0.3050 | 0.3390 | 0.2520 | 6 |
| Implementing | e | Training | -85.2000 | 1,610 | 1,220 | -1.9100 | 11.00 | 0.4620 | 0.4800 | 0.1380 | 7 |

However, in-sample validation is prone to selecting models that over-fit the data and that do not do well when predicting new data. Therefore, we performed out-of sample (OOS) validation, where values were removed from the training set and the model was evaluated on the ability to predict those missing values.

### 2. OOS Training and Testing

We computed out-of-sample accuracy of 12-month forecast from training set compared with 12-month test data, using (SWAPE)[17]

$$\begin{matrix} & SWAPE=\frac{200\times\sum_{t=firstmonth}^{t=lastmonth} \left| reported_{t}-predicted_{t} \right|}{\sum_{t=firstmonth}^{t=lastmonth} \left( \left| reported_{t} \right|+\left| predicted_{t} \right| \right)} \end{matrix}$$

where $reported_{t}$ is the actual value at month t and where $predicted_{t}$ is the predicted value at month t. The model with the lowest mean SWAPE across both the implementing and comparison groups was selected to generate the predicted numbers of cases during the intervention period.

**Table S26.** OOS validation results

| Intervention | Model | SWAPE | Intervention Rank | Percent Diff | Mean of Comparison and Intervention |
| --- | --- | --- | --- | --- | --- |
| Comparison | AN | 7.383 | 1 | 0.0 | 7.520055 |
| Comparison | ANT | 7.476 | 2 | 1.3 | 7.063058 |
| Comparison | AT | 8.395 | 3 | 13.7 | 8.396559 |
| Comparison | P8 | 8.951 | 4 | 21.2 | 9.230729 |
| Comparison | NT | 9.524 | 5 | 29.0 | 8.067032 |
| Comparison | N | 9.667 | 6 | 30.9 | 9.686032 |
| Comparison | P4 | 11.142 | 7 | 50.9 | 10.646817 |
| Comparison | P1 | 11.425 | 8 | 54.7 | 10.646514 |
| Comparison | A | 11.672 | 9 | 58.1 | 11.181656 |
| Comparison | AENT | 13.057 | 10 | 76.9 | 14.704455 |
| Implementing | NT | 6.610 | 1 | 0.0 | 8.067032 |
| Implementing | ANT | 6.650 | 2 | 0.6 | 7.063058 |
| Implementing | AN | 7.657 | 3 | 15.8 | 7.520055 |
| Implementing | AT | 8.398 | 4 | 27.0 | 8.396559 |
| Implementing | P8 | 9.511 | 5 | 43.9 | 9.230729 |
| Implementing | nN | 9.706 | 6 | 46.8 | 9.686032 |
| Implementing | P1 | 9.868 | 7 | 49.3 | 10.646514 |
| Implementing | P4 | 10.151 | 8 | 53.6 | 10.646817 |
| Implementing | A | 10.691 | 9 | 61.7 | 11.181656 |
| Implementing | T | 13.706 | 10 | 107.4 | 13.693355 |

For the comparison group, the AN model has least SWAPE, with ANT second. For Implementing group, the NT model has the least SWAPE, and ANT is second. Overall, the ANT model has the smallest SWAPE.

**Figure S6**. SWAPE distribution from OOS validation comparison (comparator) and implementing (vaccinating) groups

## Validation Algorithm: Optimizing versus Synchronizing

When predicting outcomes across two study groups, an important methodological decision is whether to select the best-fitting model independently for each group (optimize) or to use the same model type for both groups (synchronize). Optimizing maximizes predictive accuracy within each group, but introduces a risk of bias: different model classes recover trend and seasonal components differently, so an observed difference between groups could reflect model behavior rather than a true intervention effect. Synchronizing eliminates this source of bias but may sacrifice fit if the groups have meaningfully different time-series characteristics.

We used out-of-sample (OOS) validation to guide model selection, preferring OOS over in-sample fit because models that are overfit to training data tend to perform poorly when forecasting new observations. The test set comprised the most recent 12 months of the pre-intervention period, chosen to span a full seasonal cycle. Models were ranked by the symmetric weighted absolute percentage error (SWAPE), with lower values indicating better forecast accuracy.

Where possible, we selected the same model class for both the comparison and implementing groups (synchronize). When the top-ranked models differed between groups, we examined whether their SWAPE estimates and standard errors overlapped; if a common model fell within the uncertainty bounds of the best model for each group, we selected that common model. Only when no shared model provided acceptable fit for both groups did we allow the groups to use different model classes (optimize). This approach prioritizes internal validity — ensuring that differences in predicted cases reflect the intervention rather than model choice — while retaining flexibility where the data genuinely require it.

After validating the model, we re-ran the model including all the data prior to the intervention period (Jan 2015-Dec 2019) and used that model to predict the counterfactual for 1-12 months, 1-24 months, and 1-36 months post implementation.

## Estimating Cumulative Impact: 1-12 Months Post-Implementation

**Figure S7.** Predicted (pink) and actual cases (black) during 12-month period following RTS,S implementation

**Figure S8**. Estimated difference between predicted and actual number of cases during first 12-months post implementation (2000 simulations).

**Table S28.** Results of 2000 simulations of the WPE between predicted and actual cases during 12-months post-implementation

| Model | Intervention | WPE  mean | WPE  sd |
| --- | --- | --- | --- |
| ANT | Comparison | -11.58 | 1.240 |
| ANT | Implementing | -17.20 | 0.95 |

**Figure S9.** Estimated impact during first 12-months post vaccination. (2000 simulations)

**Table S29.** Impact during first 12-months post implementation (2000 simulations).

| Evaluation Period | Iterations | Mean | sd | median | HDI  Lower | HDI  Upper |
| --- | --- | --- | --- | --- | --- | --- |
| 1-12 months | 2,000 | -5.63 | 1.6 | -5.57 | -8.93 | -2.7 |

### Minimum detectable difference

Assuming the distribution of 2 groups are same as those observed, the minimum detectable difference would be +/- 3.0%.

## Estimating Cumulative Impact: 1-24 Months Post Implementation

We start with the same model selected for year 1 counterfactual but create a new counterfactual for 1-24 months and then calculate the SWAPE across 2000 simulations.

**Figure S10.** Estimated difference between predicted and actual number of cases during first 24-months post implementation (2000 simulations).

**Table S30.** Results of 2000 simulations of the WPE between predicted and actual cases during 24-months post-implementation

| Model | Intervention | WPE  Mean | WPE  SD |
| --- | --- | --- | --- |
| ANT | Comparison | -8.32 | 1.11 |
| ANT | Implementing | -16.56 | 0.72 |

**Figure S11**. Estimated impact during 24-months post vaccination. (2000 simulations)

**Table S31.** Impact during first 24-months post implementation (2000 simulations).

| Evaluation Period | Mean | SD | Median | HDI  Lower | HDI  Upper |
| --- | --- | --- | --- | --- | --- |
| 1-24 months | -8.24 | 1.31 | -8.23 | -10.73 | -5.78 |

### Minimum detectable difference

Assuming the distribution of 2 groups are same as those observed, the minimum detectable difference would be +/- 2.0%.

## Estimating Cumulative Impact: 1-36 Months Post Implementation

We start with the same model selected for year 1 counterfactual but create a new counterfactual for 1-36 months and then calculate the SWAPE across 2000 simulations.

**Figure S12.** Estimated difference between predicted and actual number of cases during 36-months post implementation (2000 simulations).

**Table S32.** Results of 2000 simulations of the WPE between predicted and actual cases during 36-months post-implementation

| Model | Intervention | WPE  Mean | WPE  SD |
| --- | --- | --- | --- |
| ANT | Comparison | -2.21 | 1.09 |
| ANT | Implementing | -13.78 | 0.66 |

**Figure S13.** Estimated impact during 36-months post vaccination. (2000 simulations)

**Table S33**. Impact during 36-months post implementation (2000 simulations).

| Evaluation Period | Mean | SD | Median | HDI  Lower | HDI  Upper |
| --- | --- | --- | --- | --- | --- |
| 1-36 months | -11.57 | 1.27 | -11.57 | -14.20 | -9.19 |

### Minimum detectable difference

Assuming the distribution of 2 groups are same as those observed, the minimum detectable difference would be +/- 2.1%.

# **Supplement I: Impact of short distance from comparison to implementing facilities**

- We calculated the distances between each comparison and all implementing facilities (regardless of whether their data was included in the final analysis) and then selected the shortest distance for each comparison facility.

**Figure S14.** Distance (km) from comparison facility to nearest implementing facility

**Figure** 21 Distance (km) from comparison facility to nearest implementing facility

We set 5km as the cutoff between facilities that are near to an implementing facility and those that are far from an implementing facility. Geolocation was unavailable for 291 of the 840 implementing facilities and 13 of the 139 comparison facilities included in analysis that consistently reported confirmed cases.

Of those comparator facilities with geocoordinates, 83 (65.9%) were classified as far and 43 (34.1%) as near.

**Figure S15.** Map of comparator facilities classified by proximity to the nearest implementing facility. Purple lines show the path from ‘near’ comparison facilities to the closest implementing facility; red line shows the path from ‘far’ comparison facilities to the closest implementing facility.

### Impact: Implementing compared with **near** comparison facilities

**Table S34.** Results of 2000 simulations of the WPE between predicted and actual cases during 36-months post-implementation. Restricted to 43 **near** comparison facilities.

| Intervention | Model | Mean | SD | HDI  Lower | HDI  Upper |
| --- | --- | --- | --- | --- | --- |
| Comparison | ant | -6.9 | 1.3 | -9.5 | -4.3 |
| Implementing | ant | -13.8 | 0.7 | -15.1 | -12.5 |
| Difference | Implementing - Comparison | -6.9 | 1.5 | -10.0 | -4.3 |

### Impact: Implementing compared with **far** comparison facilities

**Table S35.** Results of 2000 simulations of the WPE between predicted and actual cases during 36-months post-implementation. Restricted to 83 **far** comparison facilities.

| Intervention | Model | Mean | SD | HDI  Lower | HDI  Upper |
| --- | --- | --- | --- | --- | --- |
| Comparison | ant | -2.1 | 1.2 | -4.5 | 0.1 |
| Implementing | ant | -13.8 | 0.7 | -15.1 | -12.5 |
| Difference | Implementing - Comparison | -11.7 | 1.4 | -14.5 | -9.2 |

# **Supplement J:** **Sensitivity Analysis**

Table S36 summarizes results across 14 scenarios. Each scenario is defined by a combination of dataset type (raw, cleaned, or cleaned and consistently-reporting facilities) and model assumptions (model class, ensemble composition, and selection strategy). Each row represents one scenario; the final model used in the main analysis is highlighted in bold.

**Table S36.** Sensitivity analysis results across 14 scenarios.

| **Data** | **Scenario** | **Intervention** | **Model*** | **mean** | **CI** |
| --- | --- | --- | --- | --- | --- |
| Raw | Under5 | Comparison | AENT | -6.2 | (-28.8, 14.2) |
|  |  | Implementing | AENT | -9.4 | (-23.6, 2.5) |
|  |  | Difference | Implementing - Comparison | -3.2 | (-29.1, 23.9) |
| Raw | Over4 | Comparison | EN | -65.3 | (-71.2, -59.0) |
|  |  | Implementing | EN | -70.1 | (-77.2, -62.9) |
|  |  | Difference | Implementing - Comparison | -4.8 | (-14.2, 4.6) |
| Raw | Under5  with Over4 covariate | Comparison | A | -9.9 | (-15.9, -4.2) |
|  |  | Implementing | A | 9.6 | (2.62, 16.2) |
|  |  | Difference | Implementing - Comparison | 19.5 | (10.6, 27.9) |
| Cleaned | Under5 | Comparison | AENT | 0.4 | (-9.1, 9.6) |
|  |  | Implementing | AENT | -10.2 | (-24.8, 1.9) |
|  |  | Difference | Implementing - Comparison | -10.6 | (-26.4, 6.3) |
| Cleaned | Over4 | Comparison | AEN | -63.2 | (-67.3, -59.0) |
|  |  | Implementing | AEN | -68.8 | (-76.4, -62.3) |
|  |  | Difference | Implementing - Comparison | -5.6 | (-13.7, 3.1) |
|  | Under5  with Over4 covariate | Comparison | ANTP4 | 20.2 | (17.8, 22.5) |
| Cleaned |  | Implementing | ANTP4 | 17.4 | (15.1, 19.6) |
|  |  | Difference | Implementing - Comparison | -2.8 | (-6.1, 0.4) |
| Cleaned and  consistently  reporting | Under5 | Comparison | P8 | -37.1 | (-41.2, -32.5) |
|  |  | Implementing | P8 | -54.3 | (-57.9, -51.0) |
|  |  | Difference | Implementing - Comparison | -17.1 | (-22.6, -11.7) |
| Cleaned and  consistently  reporting | Over4 | Comparison | P8 | -80.2 | (-81.6, -78.7) |
|  |  | Implementing | P8 | -85.7 | (-87.0, -84.6) |
|  |  | Difference | Implementing - Comparison | -5.5 | (-7.5, -3.7) |
| **Cleaned and  consistently  reporting** | **Under5**  **with Over4 covariate** | Comparison | ANT | -2.3 | (-4.4, -0.1) |
|  |  | Implementing | ANT | -13.8 | (-15.1, -12.5) |
|  |  | **Difference** | **Implementing - Comparison** | **-11.5** | **(-14.2, -9.2)** |
| Cleaned and  consistently  reporting | Always Reporting** | Comparison | ANT | -1.6 | (-3.8, 0.7) |
|  |  | Implementing | ANT | -12.3 | (-14.3, -10.3) |
|  |  | Difference | Implementing - Comparison | -10.6 | (-13.5, -7.7) |
| Cleaned and  consistently  reporting | Primary Models Only*** | Comparison | P8 | 3.5 | (-1.5, 8.5) |
|  |  | Implementing | P8 | -11.8 | (-14.1, -9.3) |
|  |  | Difference | Implementing - Comparison | -15.2 | (-21.2, -10.0) |
| Cleaned and  consistently  reporting | Optimized**** | Comparison | AN | -2.7 | (-5.35, 0.0599) |
|  |  | Implementing | NT | -19.0 | (-20.3, -17.5) |
|  |  | Difference | Implementing - Comparison | -16.3 | (-19.4, -13.2) |
| Cleaned and  consistently  reporting | Near Comparison | Comparison | ANT | -6.9 | (-9.5, -4.3) |
|  |  | Implementing | ANT | -13.8 | (-15.1, -12.5) |
|  |  | Difference | Implementing - Comparison | -6.9 | (-10.0, -4.3) |
| Cleaned and  consistently  reporting | Far Comparison | Comparison | ANT | -2.1 | (-4.5, 0.1) |
|  |  | Implementing | ANT | -13.8 | (-15.1, -12.5) |
|  |  | Difference | Implementing - Comparison | -11.7 | (-14.5, -9.2) |

*Ensemble models indicate combinations of ARIMA (A), ETS (E), NNETAR (N), Prophet-4 Fourier terms (P4), Prophet with 8 Fourier terms (P8), and TSLM (T) models.

**Include facilities that reported all months (in contrast to the final model included facilities that reported at least 11 of 12 months each year).

***No ensemble models used

****Allowed selection of model to be different for comparison and implementing groups

**Legend: The final model selected for the main analysis and reported in the main manuscript is highlighted in bold.**

**Figure S16.** Forest plot of estimates from 14 scenarios defined by different datasets and model assumptions. Points represent estimated effects and horizontal lines indicate 95% confidence intervals.

**Legend: Points represent estimates and horizontal lines indicate 95% confidence intervals. The final model reported in the main manuscript is highlighted.**

# **References**

1. RStudio Team. RStudio: Integrated Development Environment for R. Boston, MA: RStudio, PBC.; 2020.

2. Rts T, Partnership (2014) SCT. [Efficacy and Safety of the RTS,S/AS01 Malaria Vaccine during 18 Months after Vaccination: A Phase 3 Randomized, Controlled Trial in Children and Young Infants at 11 African Sites](https://doi.org/10.1371/journal.pmed.1001685). PLOS Medicine. 2014;11:e1001685.

3. RTS SCTP. [Efficacy and safety of RTS,S/AS01 malaria vaccine with or without a booster dose in infants and children in Africa: Final results of a phase 3, individually randomised, controlled trial](https://doi.org/10.1016/S0140-6736(15)60721-8). The Lancet. 2015;386:31–45.

4. Introduction - DHIS2 Documentation. https://docs.dhis2.org/en/develop/using-the-api/dhis-core-version-240/introduction.html;

5. Organisation Units - DHIS2 Documentation. https://docs.dhis2.org/en/implement/database-design/organisation-units.html;

6. List of hospitals in Kenya. Wikipedia. 2024;

7. Moturi AK, Suiyanka L, Mumo E, Snow RW, Okiro EA, Macharia PM. [Geographic accessibility to public and private health facilities in Kenya in 2021: An updated geocoded inventory and spatial analysis](https://doi.org/10.3389/fpubh.2022.1002975). Frontiers in Public Health. 2022;10.

8. Martin JL, Mosha FW, Lukole E, Rowland M, Todd J, Charlwood JD, et al. [Personal protection with PBO-pyrethroid synergist-treated nets after 2 years of household use against pyrethroid-resistant Anopheles in Tanzania](https://doi.org/10.1186/s13071-021-04641-5). Parasites & Vectors. 2021;14:150.

9. Cheng J, Schloerke B, Karambelkar B, Xie Y. Leaflet: Create interactive web maps with the JavaScript ’leaflet’ library. 2024; Available from: <https://CRAN.R-project.org/package=leaflet>

10. Wang E, Cook D, Hyndman RJ. A new tidy data structure to support exploration and modeling of temporal data. 2020;29:466–78. Available from: <https://doi.org/10.1080/10618600.2019.1695624>

11. Chapter 5.8 evaluating point forecast accuracy | forecasting: Principles and practice (3rd ed) [Internet]. Available from: <https://otexts.com/fpp3/accuracy.html>

12. Chapter 5.10 time series cross-validation | forecasting: Principles and practice (3rd ed) [Internet]. Available from: <https://otexts.com/fpp3/tscv.html>

13. Hyndman RJ, Athanasopoulos G. Forecasting: Principles and practice, 3rd edition. This online version of the book was last updated on 22 March 2024. OTexts;

14. 5.1 a tidy forecasting workflow | forecasting: Principles and practice (3rd ed) [Internet]. Available from: <https://otexts.com/fpp3/a-tidy-forecasting-workflow.html>

15. Hyndman RJ. Forecasting ensembles and combinations. https://pkg.robjhyndman.com/combinations_talk/ensembles_uscdc_2022.html#/title-slide; 2022.

16. 5.1 a tidy forecasting workflow | forecasting: Principles and practice (3rd ed) [Internet]. Available from: <https://otexts.com/fpp3/a-tidy-forecasting-workflow.html>

17. Hewamalage H, Ackermann K, Bergmeir C. Forecast Evaluation for Data Scientists: Common Pitfalls and Best Practices.

18. Linden A, Adams JL, Roberts N. [Evaluating Disease Management Program Effectiveness: An Introduction to Time-Series Analysis](https://doi.org/10.1089/109350703322682559). Disease Management. 2003;6:243–55.

19. Kolassa S. Answer to "what are the shortcomings of the mean absolute percentage error (MAPE)?" [Internet]. 2017. Available from: <https://stats.stackexchange.com/a/299713>

20. Kolassa S. [Why the “best” point forecast depends on the error or accuracy measure](https://doi.org/10.1016/j.ijforecast.2019.02.017). International Journal of Forecasting. 2020;36:208–11.

21. Kolassa S. Answer to "RMSE or MAPE? Which one to choose for accuracy?" [Internet]. 2021. Available from: <https://stats.stackexchange.com/a/554440>

22. Taylor SJ, Letham B. Forecasting at scale. The American Statistician. 2018;72(1):37–45
